# Underlining the Molecular Mechanism of Nonalcoholic Fatty Liver Disease and Coronary Artery Disease in Lipid Metabolism by Combining Multiple Sets of Data Sets

**DOI:** 10.1002/iub.70040

**Published:** 2025-07-09

**Authors:** Wei Zheng, Shouhao Wang, Huafang Wang, Chengan Xu, Qiaoqiao Yin, Hua Di

**Affiliations:** ^1^ Center for General Practice Medicine, Department of Infectious Diseases, Zhejiang Provincial People's Hospital (Affiliated People's Hospital) Hangzhou Medical College Hangzhou Zhejiang China; ^2^ Department of Infectious Diseases The First Affiliated Hospital of Wenzhou Medical University Wenzhou Zhejiang Province China; ^3^ Cancer Center, Department of Hematology Zhejiang Provincial People's Hospital (Affiliated People's Hospital), Hangzhou Medical College Hangzhou Zhejiang China; ^4^ Geriatric Medicine Center, Department of Acupuncture & Massage Zhejiang Provincial People's Hospital (Affiliated People's Hospital), Hangzhou Medical College Hangzhou Zhejiang China

**Keywords:** bioinformatics, coronary artery disease, lipid metabolism genes, nonalcoholic fatty liver disease

## Abstract

Nonalcoholic fatty liver disease (NAFLD) is closely associated with coronary artery disease (CAD); however, their shared genetic traits and molecular mechanisms in lipid metabolism remain unclear. In this study, we identified that the differentially expressed genes in NAFLD and CAD intersected with lipid metabolism genes to obtain three key genes—GPD1, MVK, and PIK3R2. Data from the GeneCards database indicated a significant correlation between NAFLD‐related regulatory genes and the expression levels of these key genes. Notably, GPD1 showed a significant positive correlation with PNPLA3 (*r* = 0.715), while PIK3R2 exhibited a significant negative correlation with MIR21 (*r* = −0.691). Similarly, CAD regulatory genes were significantly correlated with the expression levels of these key genes; GPD1 showed a significant positive correlation with APOA1 (*r* = 0.751), and PIK3R2 had a significant negative correlation with LPA (*r* = −0.362). Additionally, single‐cell sequencing analysis of NAFLD showed that GPD1, MVK, and PIK3R2 had higher activity in cells with a high expression of bile acid metabolism genes in the immune pathway. In CAD, GPD1 showed higher activity in cells with high oxidative phosphorylation in the immune pathway. Finally, we found that one drug interacted with MVK, while 38 drugs interacted with PIK3R2. This study highlights GPD1, MVK, and PIK3R2 as key genes involved in NAFLD, CAD, and lipid metabolism, suggesting potential targets for further mechanistic studies and novel therapeutic approaches for patients with NAFLD and CAD.

## 1 Introduction

Nonalcoholic fatty liver disease (NAFLD) is a common liver disease characterized by excessive lipid accumulation in the liver, leading to liver damage [1]. NAFLD is recognized as a significant public health concern worldwide. Research indicates that coronary artery disease (CAD) is the leading cause of death among patients with NAFLD, alongside liver and extrahepatic malignancies [2, 3]. The occurrence of these two diseases arises from complex interactions among multiple genetic variations and environmental factors, such as dyslipidemia, obesity, and oxidative stress, although the complete pathogenesis is not fully understood [4, 5]. Additionally, genetic polymorphisms have been shown to influence individuals' susceptibility to NAFLD and CAD [6, 7].

Advancements in bioinformatics and gene chip technology have allowed researchers to gain deeper insights into the genetic basis of disease pathogenesis. In this study, we employed bioinformatics analysis to identify the common pathways and key genes involved in NAFLD and CAD, aiming to uncover shared mechanisms and therapeutic targets. These findings could serve as a basis for improved early diagnosis and treatment of patients with NAFLD and CAD.

## 2 Methods

### 2.1 Data Download

The Gene Expression Omnibus (GEO) database, hosted by the National Center for Biotechnology Information (NCBI), serves as a rich source for gene expression data. The GSE89632 dataset contains expression profiles from 63 samples (24 controls and 39 disease cases), annotated with the GPL14951 platform. Likewise, the GSE113079 dataset provides expression data for 141 samples (48 controls and 93 disease cases), annotated on the GPL20115 platform. Additionally, single‐cell RNA sequencing data were collected, including 40 case profiles from GSE202379 and 4 cases from GSE121893 for further exploration.

### 2.2 Differential Expression Analysis

We utilized the Limma package in R for differential gene expression analysis, identifying significant genes between control and disease groups to investigate the molecular underpinnings of NAFLD and CAD comorbidity. Genes with |logFC| > 0.585 and *p* < 0.05 were considered significant, and results were visualized using volcano plots and heat maps.

### 2.3 Functional Enrichment Analysis

Metascape (www.metascape.org) was employed to annotate overlapping genes, providing insights into functional relationships. Gene Ontology (GO) pathway enrichment analysis was performed, with a minimum overlap of ≥ 3 and a *p*‐value ≤ 0.01 for statistical significance.

### 2.4 WGCNA Analysis

A weighted gene co‐expression network was built to identify co‐expressed gene modules and their associations with disease phenotypes. Using the WGCNA package, we transformed the weighted adjacency matrix into a topological overlap matrix (TOM) to assess connectivity. Hierarchical clustering was applied to construct a dendrogram, organizing genes into modules based on expression similarities, with distinct modules color‐coded.

### 2.5 Immune Cell Infiltration Analysis

The CIBERSORT method was used to estimate immune cell populations within the sample microenvironment. Utilizing support vector regression, CIBERSORT deconvolutes expression data to infer the proportions of 22 immune cell phenotypes, including T cells, B cells, plasma cells, and myeloid subsets. Correlations between immune cell content and gene expression were subsequently analyzed.

### 2.6 Gene Set Enrichment Analysis

To compare signaling pathways between groups with high and low gene expression, gene set enrichment analysis (GSEA) was performed. The background gene sets, obtained from the MsigDB (Version 7.0), were analyzed for differential pathway expression, with enriched gene sets ranked by their enrichment scores (adjusted *p*‐value < 0.05). GSEA is commonly used to integrate disease classification with biological significance.

### 2.7 Gene Set Variation Analysis

Gene set variation analysis (GSVA), a non‐parametric, unsupervised method, was utilized to evaluate gene set enrichment across samples. Gene‐level variations were translated into pathway‐level changes, and gene sets from the Molecular Signatures Database were scored to assess potential biological function changes across samples.

### 2.8 Transcriptional Regulation Analysis of Key Genes

The RcisTarget package was employed for predicting transcription factors, based on motif analysis. Normalized enrichment scores (NES) for each motif were computed, and additional annotations were generated using motif similarity and gene sequence data. Motif overrepresentation in gene sets was assessed by calculating the area under the curve (AUC) for each motif‐gene pair.

### 2.9 Single Cell Analysis

Single‐cell expression profiles were analyzed using the Seurat package. Low‐expression genes were filtered, followed by standardization, normalization, principal component analysis (PCA), and harmony analysis. UMAP was applied to visualize cluster relationships, and clusters were annotated using the Celldex package to focus on cells critical to disease progression.

### 2.10 Drug Prediction

The Drug‐Gene Interaction Database (DGIdb) was used to identify potential drugs and molecular compounds interacting with key genes. DGIdb integrates data on drug targets, drug‐metabolizing enzymes, and drug‐gene associations, facilitating the exploration of gene‐drug interactions for personalized medicine and drug development.

### 2.11 Statistical Analysis

All statistical analyses were performed using R software (Version 4.2.2). A *p*‐value of < 0.05 was considered statistically significant, and all tests were two‐sided.

## 3 Results

### 3.1 Differential Expression and Pathway Enrichment Analysis

**FIGURE 1:**
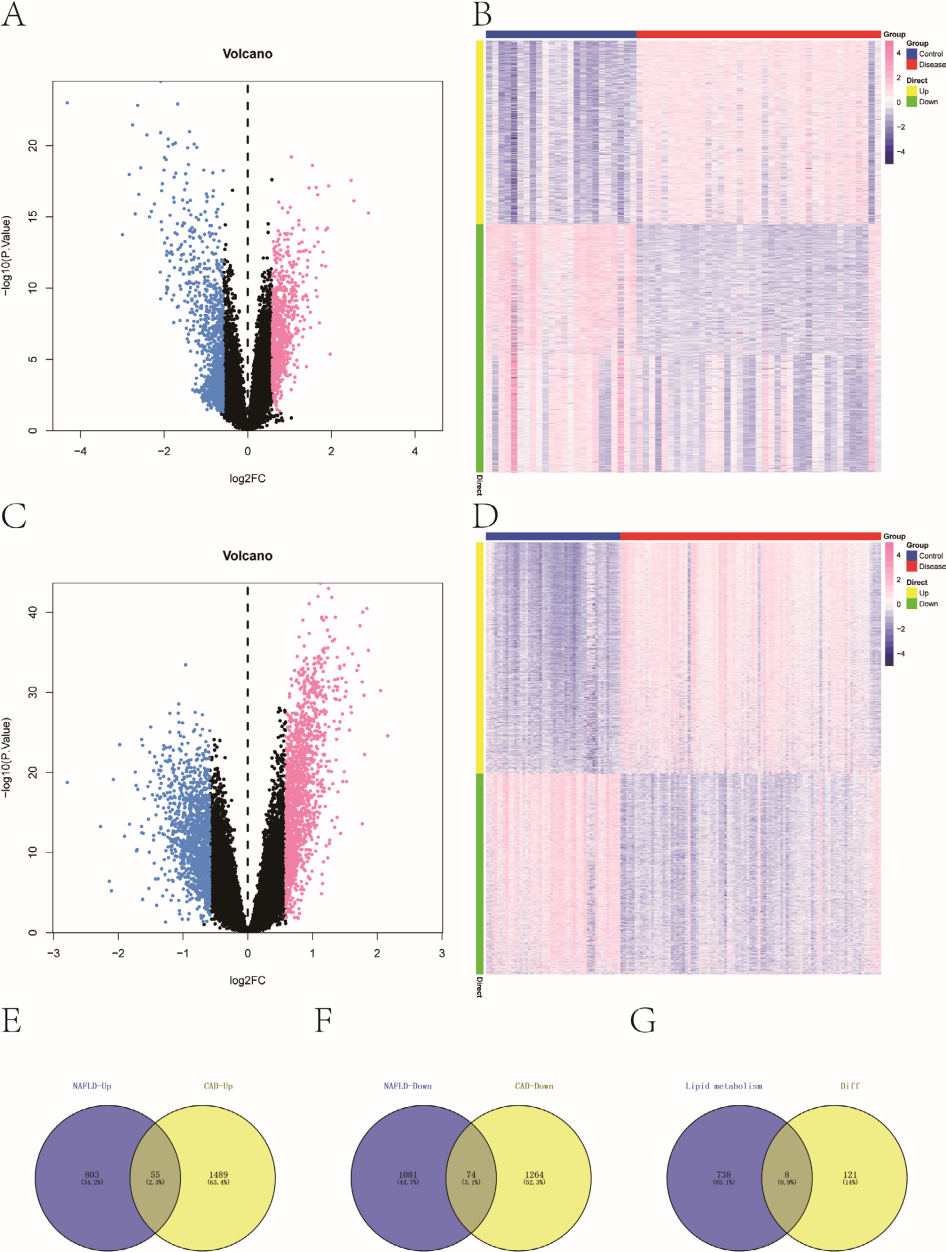
(A, B) Differential genes of NAFLD patients in GSE89632 dataset. (C, D) Differential genes of CAD patients in GSE113079 dataset. (E) Differentially expressed up‐regulated genes between NAFLD and CAD patients. (F) Differentially expressed down‐regulated genes between NAFLD and CAD patients. (G) The intersection genes between lipid metabolism genes and differential genes.

We retrieved the GSE89632 dataset from the NCBI GEO database, which includes gene expression profiles from 63 NAFLD samples—24 from controls and 39 from the disease group. Using the Limma package, we identified differentially expressed genes (DEGs) based on a threshold of *p* < 0.05 and |logFC| > 0.585. This yielded 2013 DEGs, of which 858 were upregulated and 1155 were downregulated (Figure 1A,B). Similarly, the GSE113079 dataset, containing 141 CAD samples (48 controls, 93 disease cases), was analyzed with the same method, revealing 2882 DEGs—1544 upregulated and 1338 downregulated (Figure 1C,D).

We found 55 genes upregulated and 74 genes downregulated in both datasets (Figure 1E,F). Furthermore, we extracted 746 lipid metabolism‐related genes from the Molecular Signatures Database and identified eight overlapping genes by intersecting them with the DEGs (Figure 1G). Metascape was employed to conduct pathway enrichment analysis, identifying key associations with lipid biosynthesis, organophosphate biosynthesis, and alcohol metabolism (Figure S1).

### 3.2 Weighted Gene Co‐Expression Networks

**FIGURE 2:**
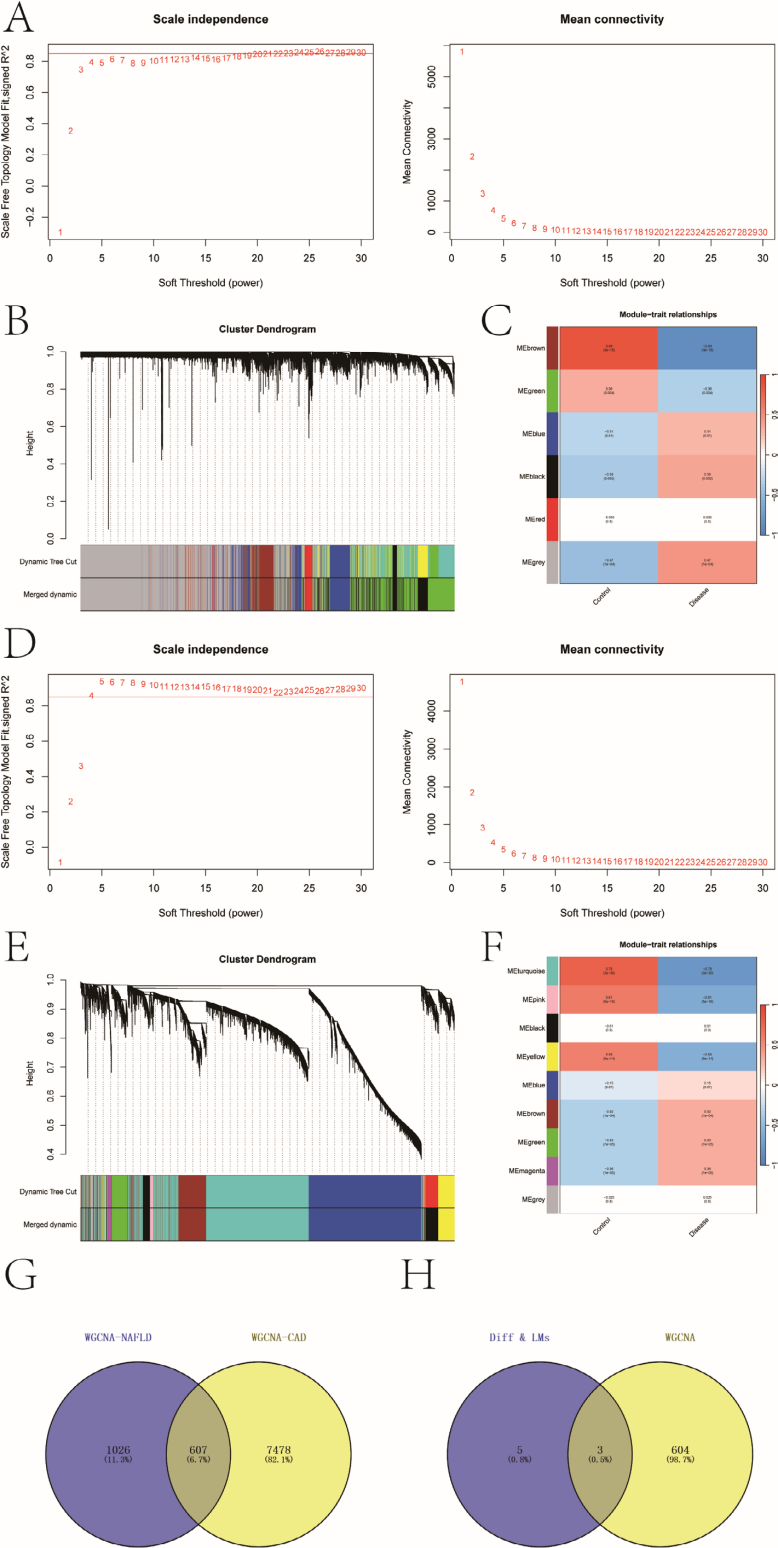
(A) The soft threshold *β* in GSE89632 dataset. (B, C) The module genes based on tom matrix detection. (D) The soft threshold *β* in GSE113079 dataset. (E, F) The module genes based on tom matrix detection. (G) The intersection genes between the two datasets module genes. (H) The key genes between intersection genes and differential intersection genes of lipid metabolism.

To detect key genes in NAFLD and CAD, we constructed WGCNA based on the expression profiles. In the GSE89632 dataset, we set the soft threshold (*β*) to 23 (Figure 2A) and identified six gene modules through TOM (Figure 2B,C), with the brown module exhibiting the highest disease correlation (cor = −0.84, *p* = 4e−18). In GSE113079, we applied a soft threshold of *β* = 4 (Figure 2D), identifying nine gene modules (Figure 2E,F), where the turquoise module had the strongest association with CAD (OR = −0.78, *p* = 2 × 10^−30^). We then intersected the most correlated modules from both datasets, resulting in 607 overlapping genes (Figure 2G). Cross‐referencing these with the lipid metabolism‐related genes yielded three core genes: GPD1, MVK, and PIK3R2, which were prioritized for further investigation (Figure 2H).

### 3.3 NAFLD Immune Infiltration Analysis

**FIGURE 3:**
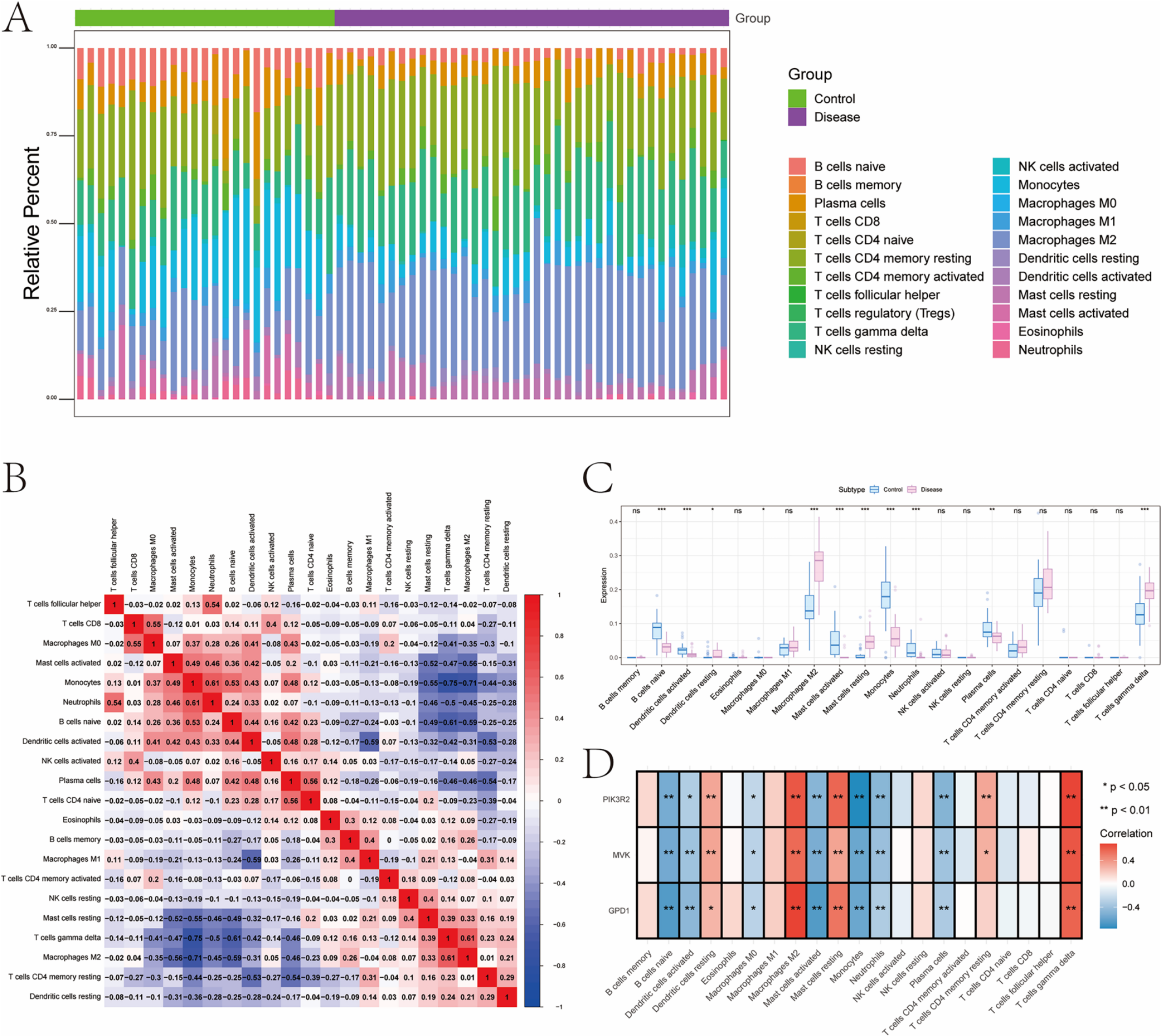
(A–C) The correlation between the distribution of immune infiltration level and immune cells in patients with NAFLD. (D) In patients with NAFLD, the correlation between key genes and immune cells.

The microenvironment, comprising immune cells, fibroblasts, extracellular matrix, and growth/inflammatory factors, plays a crucial role in disease progression, prognosis, and treatment response. We analyzed immune cell infiltration and their correlations, showing that resting dendritic cells and M2 macrophages were elevated in the disease group, while activated dendritic cells and naive B cells were reduced (Figure 3A,C). Correlation analysis revealed that GPD1 was positively associated with gamma delta T cells and M2 macrophages, while negatively correlated with naive B cells and activated mast cells. Similarly, MVK and PIK3R2 exhibited comparable correlations with these immune cells (Figure 3D).

### 3.4 CAD Immune Infiltration Analysis

**FIGURE 4:**
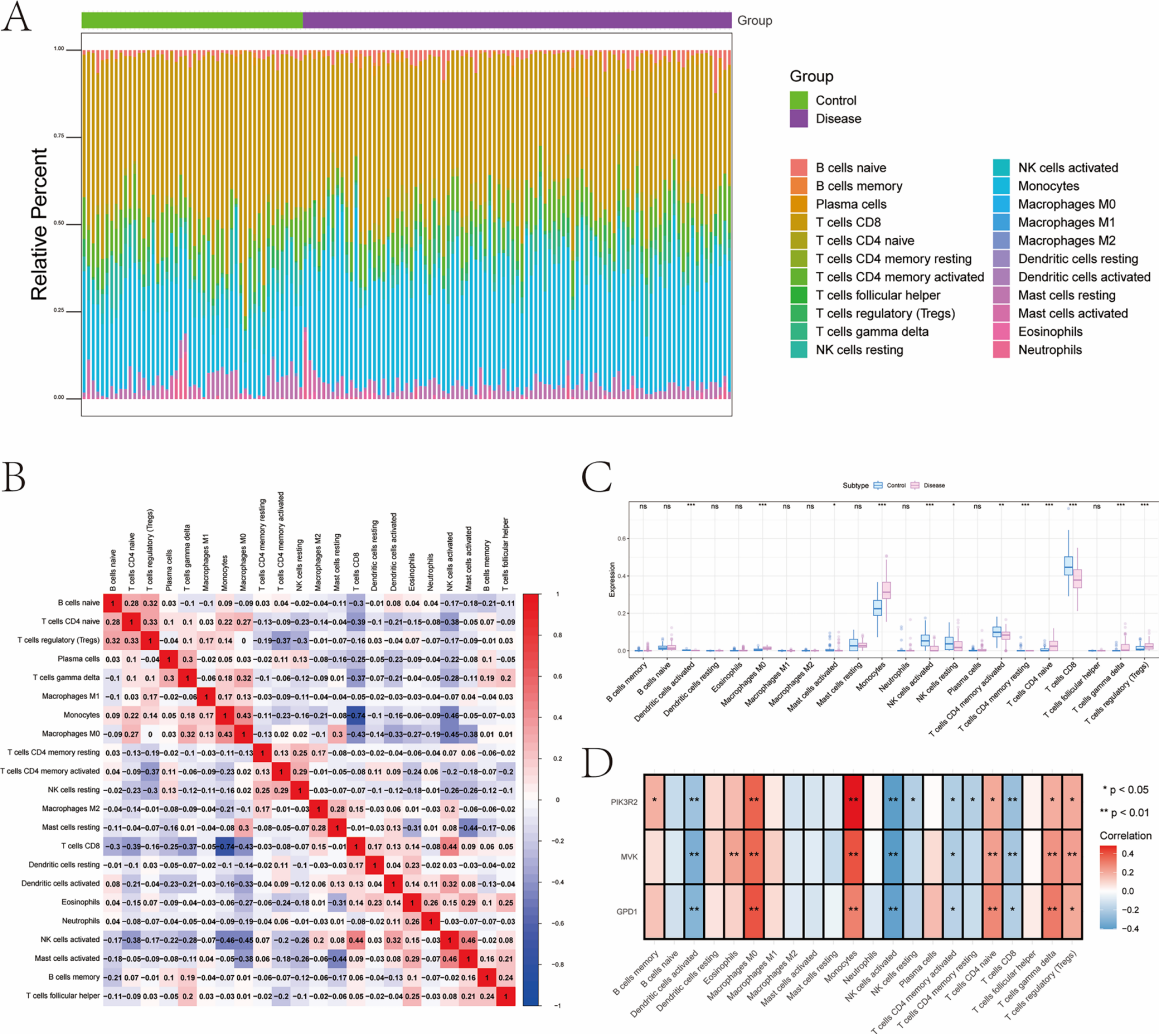
(A–C) The correlation between the distribution of immune infiltration level and immune cells in patients with CAD. (D) In patients with CAD, the correlation between key genes and immune cells.

Immune infiltration was assessed in CAD, revealing increased levels of regulatory T cells (Tregs) and monocytes, with a reduction in activated mast cells and CD8 T cells in the disease group (Figure 4A,C). Correlation analysis showed that GPD1 and MVK were positively correlated with M0 macrophages and monocytes, while PIK3R2 was also linked to these cell types but negatively correlated with activated NK cells and CD8 T cells (Figure 4D). Further exploration using TISIDB revealed significant associations between the key genes and various immune factors, indicating their important roles in modulating the immune microenvironment (Figures S2 and S3).

### 3.5 GSEA Analysis

**FIGURE 5:**
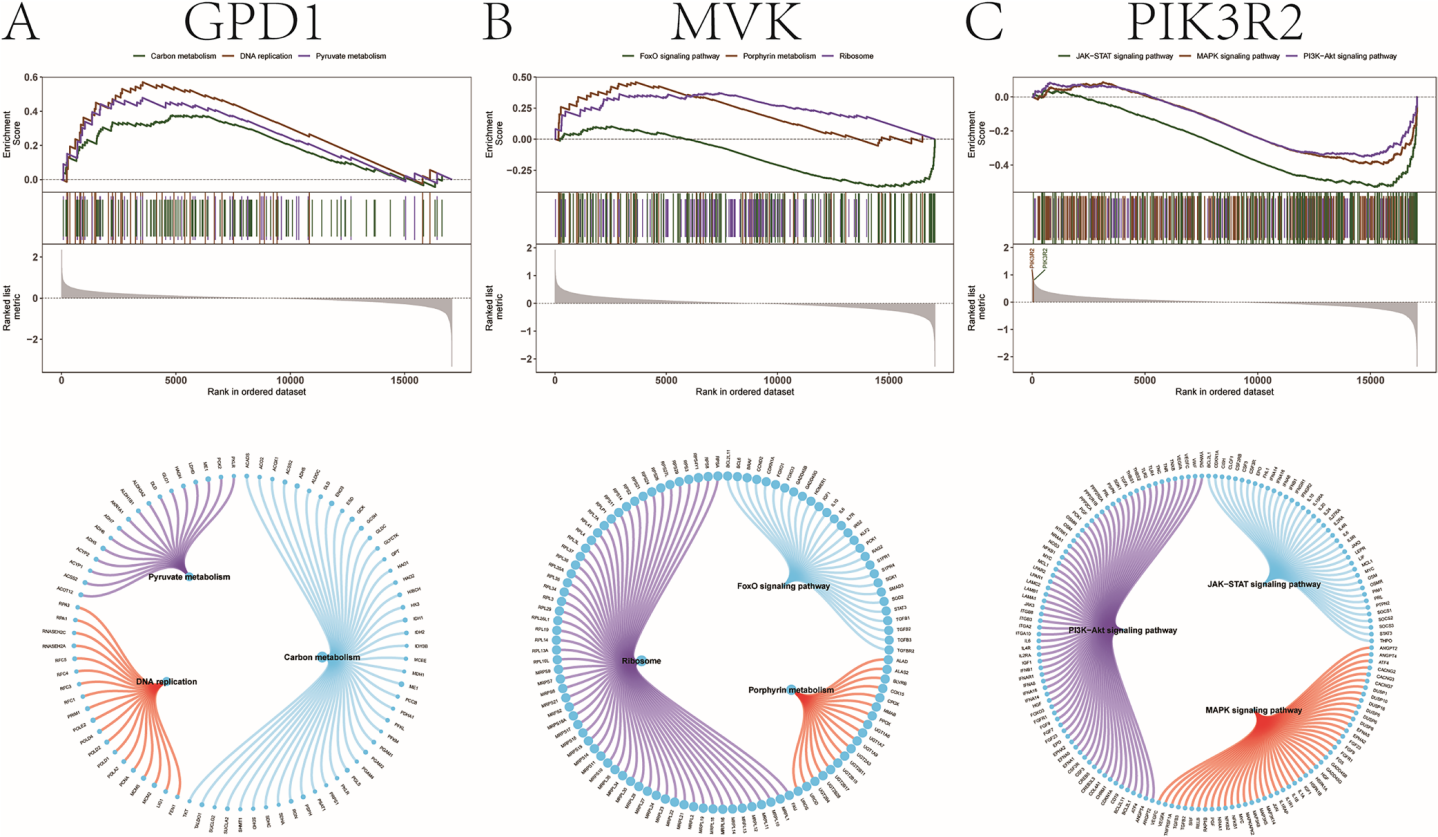
(A) The potential molecular mechanism of key gene GPD1 affecting the progression of NAFLD. (B) The potential molecular mechanism of the key gene MVK affecting the progression of NAFLD. (C) The potential molecular mechanism of key gene PIK3R2 affecting the progression of NAFLD.

**FIGURE 6:**
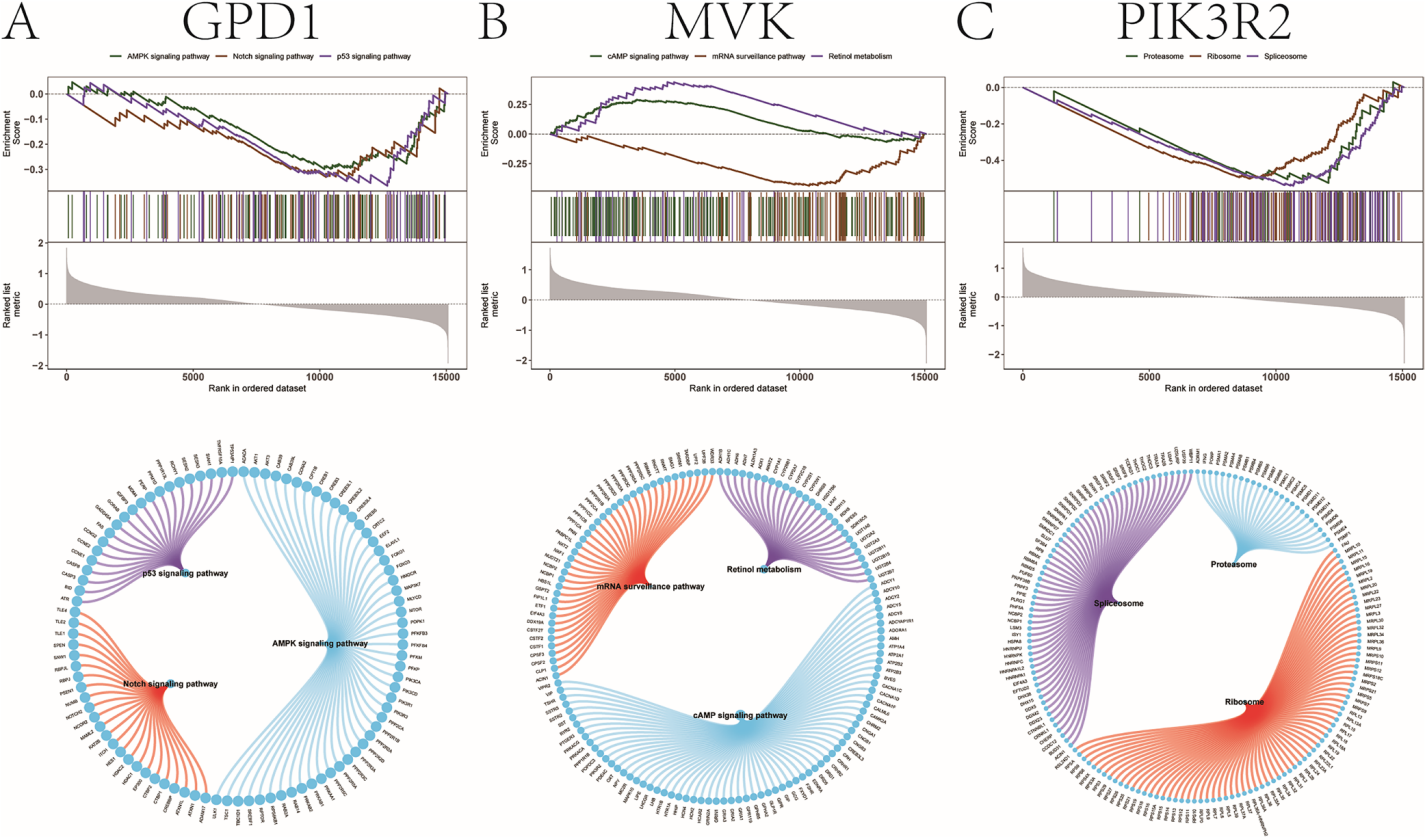
(A) The potential molecular mechanism of key gene GPD1 affecting the progression of NAFLD. (B) The potential molecular mechanism of the key gene MVK affecting the progression of NAFLD. (C) The potential molecular mechanism of key gene PIK3R2 affecting the progression of NAFLD.

GSEA was performed to elucidate the pathways linked to the key genes. For NAFLD, GPD1 was enriched in pathways such as DNA replication and carbon metabolism (Figure 5A), while MVK showed enrichment in the FoxO signaling and porphyrin metabolism pathways (Figure 5B). PIK3R2 was associated with the PI3K‐Akt, JAK–STAT, and MAPK pathways (Figure 5C). For CAD, GPD1 was enriched in p53, AMPK, and Notch pathways (Figure 6A), while MVK and PIK3R2 were linked to mRNA surveillance and proteasome pathways, respectively (Figure 6B,C).

### 3.6 GSVA Analysis

**FIGURE 7:**
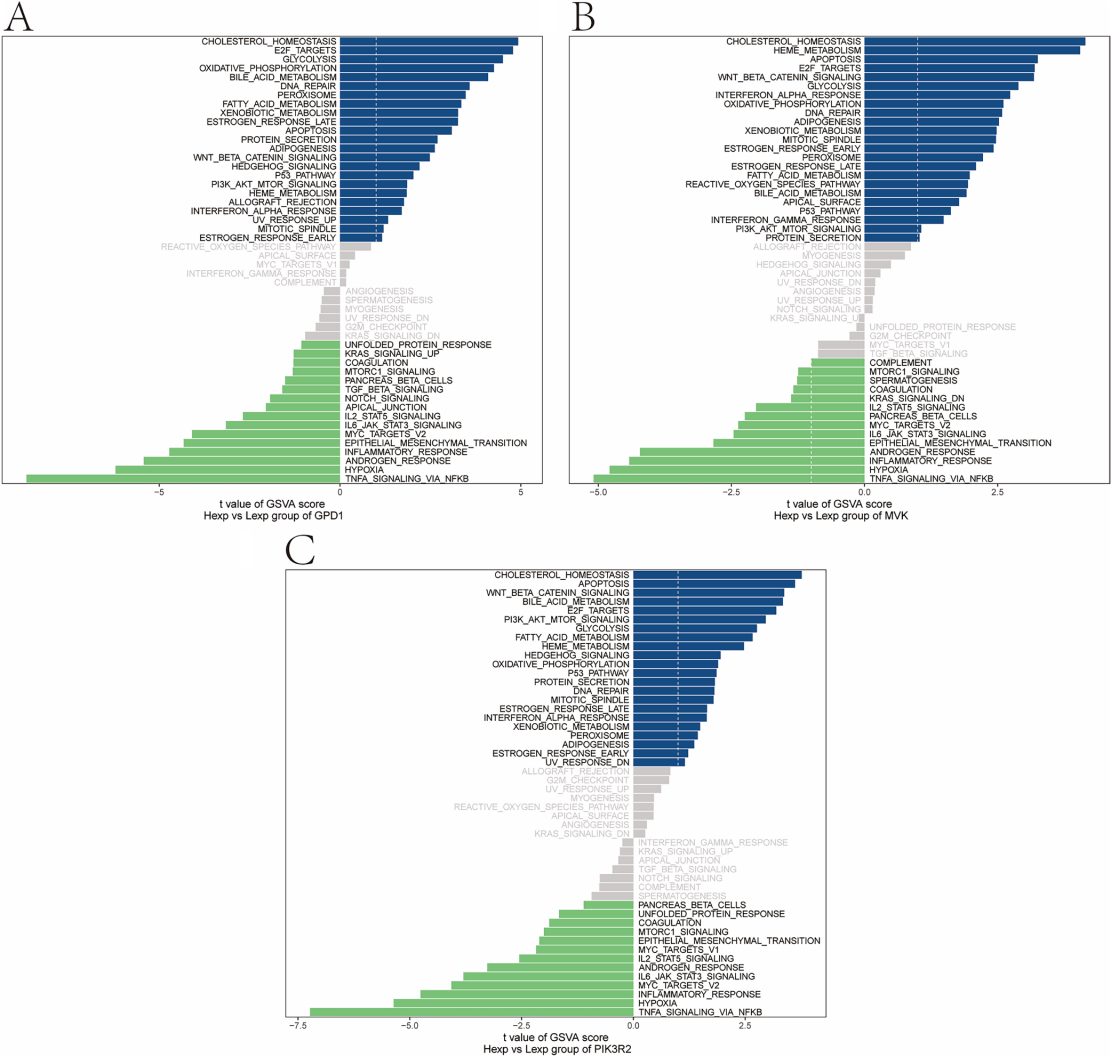
The results of GSVA analysis of NAFLD. (A) The key gene GPD1 enriched signaling pathway. (B) The key gene MVK enriched signaling pathway. (C) The key gene PIK3R2 enriched signaling pathway.

**FIGURE 8:**
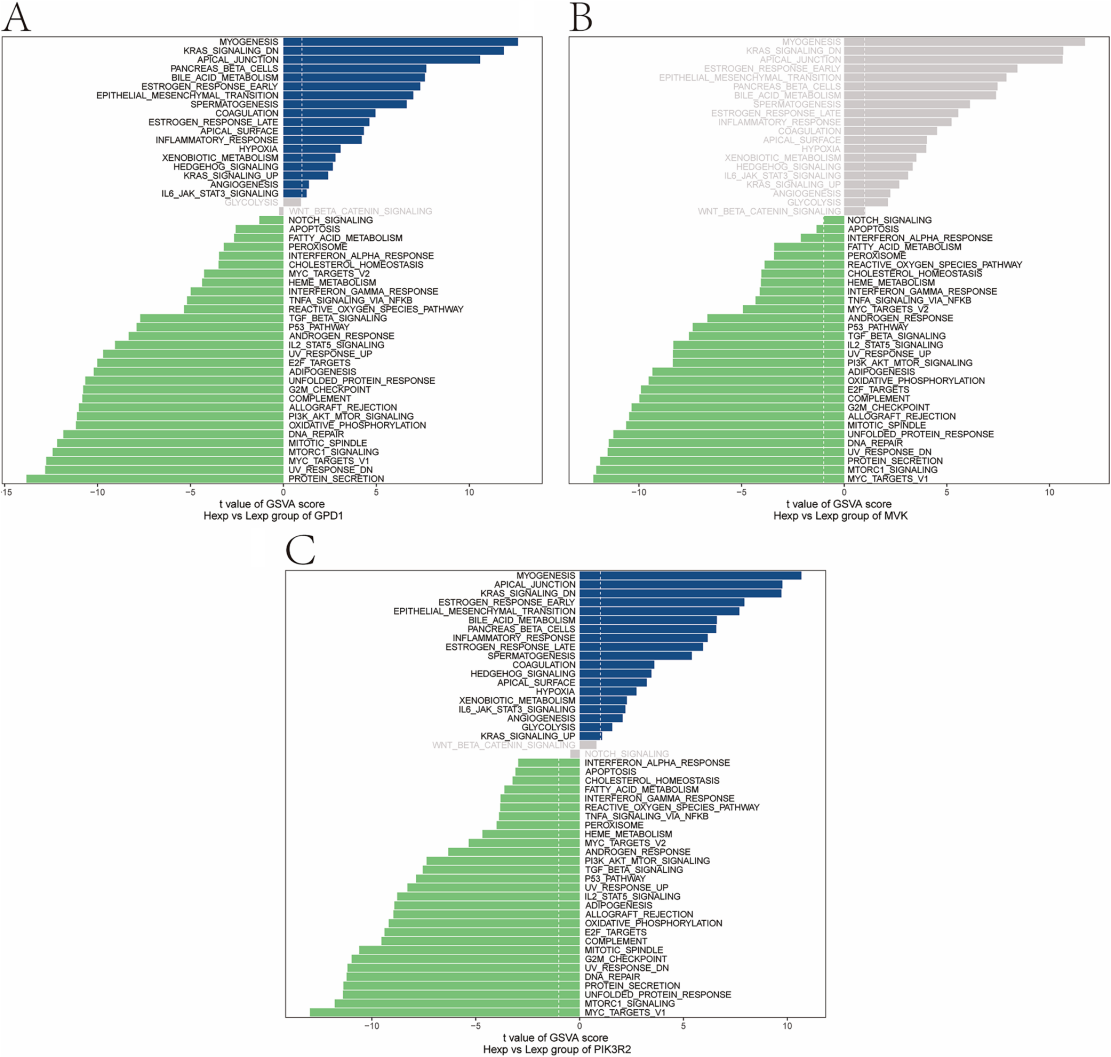
The results of GSVA analysis of CAD. (A) The key gene GPD1 enriched signaling pathway. (B) The key gene MVK enriched signaling pathway. (C) The key gene PIK3R2 enriched signaling pathway.

GSVA was applied to assess gene set variation at the transcriptome level. For NAFLD, GPD1 was linked to CHOLESTEROL_HOMEOSTASIS and E2F_TARGETS (Figure 7A), while MVK was enriched in MYOGENESIS and KRAS_SIGNALING_DN pathways (Figure 7B). PIK3R2 was associated with pathways like APOPTOSIS (Figure 7C). For CAD, similar patterns were observed, with GPD1 linked to cholesterol homeostasis and PIK3R2 connected to pathways like MYOGENESIS and APICAL_JUNCTION (Figure 8A,C).

### 3.7 Transcriptional Regulation Analysis of Key Genes

Using the RcisTarget package, we conducted motif enrichment analysis to identify transcription factors regulating the key genes. The motif with the highest normalized enrichment score (NES: 6.72) was cisbp__M0462. We present the enriched motifs and corresponding transcription factors for these genes (Figure S4).

### 3.8 Metabolic Pathway Correlation

To explore the connection between key genes and metabolic pathways, we quantified pathway activity in NAFLD and CAD expression profiles using ssGSEA and visualized the results through heatmaps (Figure S5A,B). In NAFLD, lipid metabolism and drug metabolism pathways were significantly upregulated in the disease group, while in CAD, lipid metabolism signatures were prominent. Correlations between key genes and metabolic pathways were displayed as Tables S1 and S2.

### 3.9 Disease Correlation Analysis of Key Genes

**FIGURE 9:**
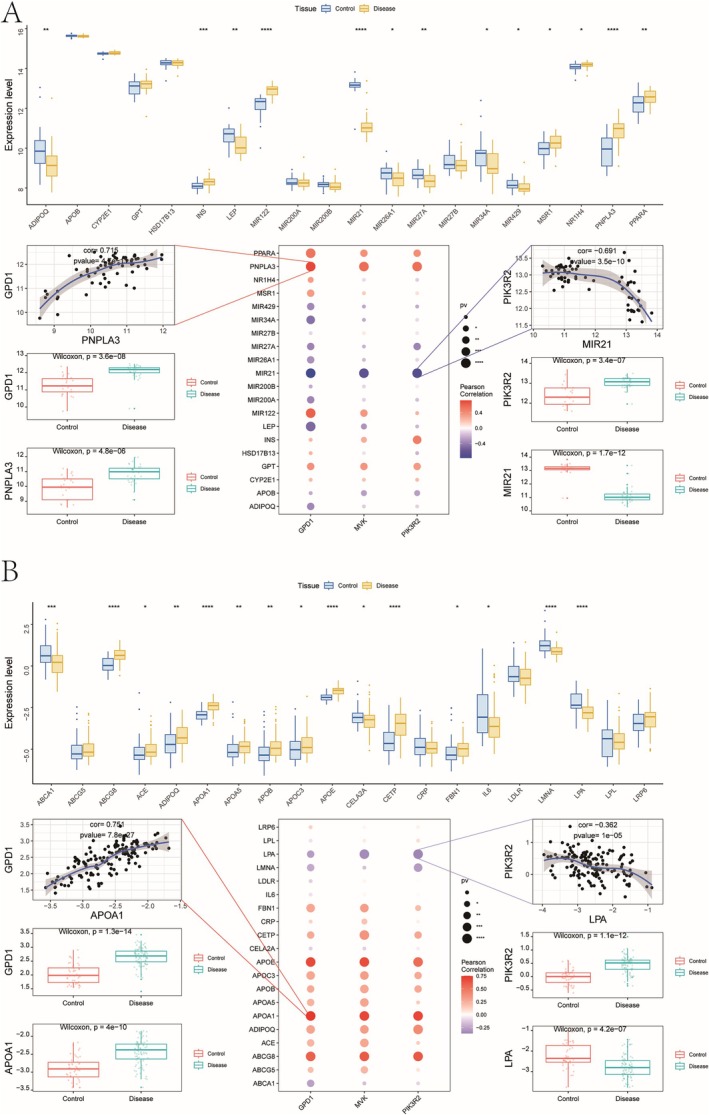
(A) In the GeneCards database, NAFLD disease regulation related gene expression differences between groups. (B) In the GeneCards database, the differences in the expression of disease‐related genes in CAD between groups.

Using the GeneCards database, we retrieved the top 20 disease‐related genes for NAFLD and CAD. Expression analysis showed significant differences between the control and disease groups (Figure 9A,B). Correlation analysis highlighted key associations, with GPD1 showing a positive correlation with PNPLA3 (*r* = 0.715) in NAFLD, and PIK3R2 negatively correlated with MIR21 (*r* = −0.691). Similar trends were observed in CAD, with GPD1 correlating positively with APOA1 (*r* = 0.751) and PIK3R2 negatively with LPA (*r* = −0.362).

### 3.10 Single‐Cell Analysis of NAFLD

**FIGURE 10:**
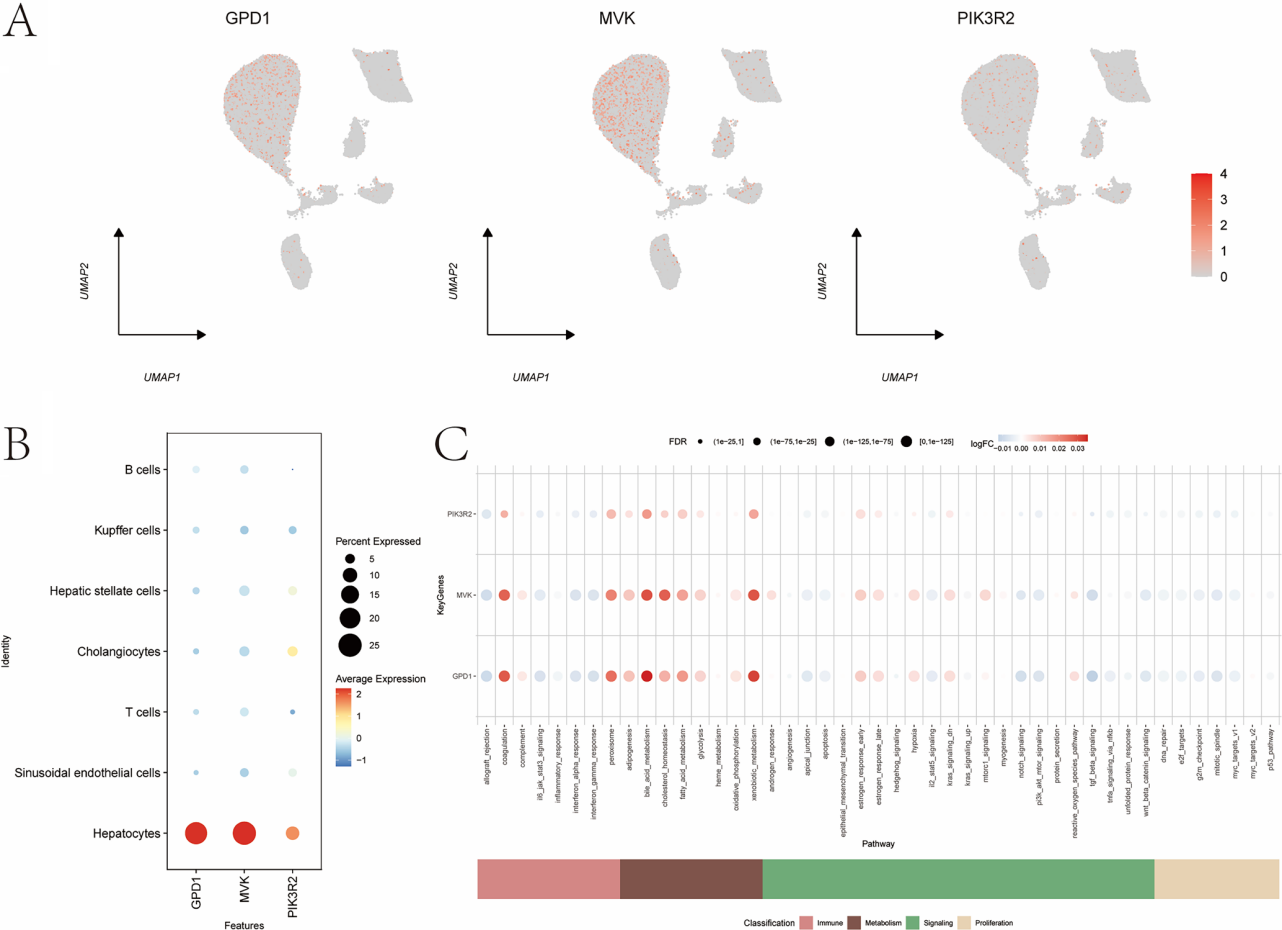
(A, B) The expression of key genes in single cell. (C) The expression differences of key genes in immune metabolism related pathways.

**FIGURE 11:**
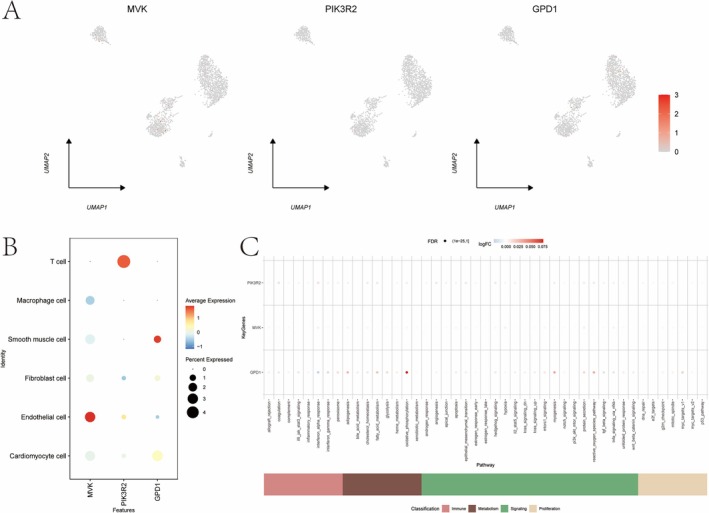
(A, B) The expression of key genes in single cell. (C) The expression differences of key genes in immune metabolism related pathways.

The Seurat package was used to process NAFLD single‐cell data (Figures 10 and 11). Following filtering, normalization, PCA, and UMAP clustering, 12 cell subtypes were identified and categorized into hepatocytes, sinusoidal endothelial cells, T cells, and others (Figure S6). Co‐expression analysis revealed that GPD1, MVK, and PIK3R2 were highly expressed in bile acid metabolic pathways, especially in specific cell types (Figures S7–S9).

### 3.11 Single‐Cell Analysis of CAD

Similar methods were applied to CAD single‐cell data, identifying six subtypes, including cardiomyocytes and macrophages (Figure S10). Co‐expression analysis demonstrated that GPD1 exhibited elevated activity in cells with higher oxidative phosphorylation in immune pathways (Figure S11–S13).

### 3.12 Targeted Therapy Drug Prediction

**FIGURE 12:**
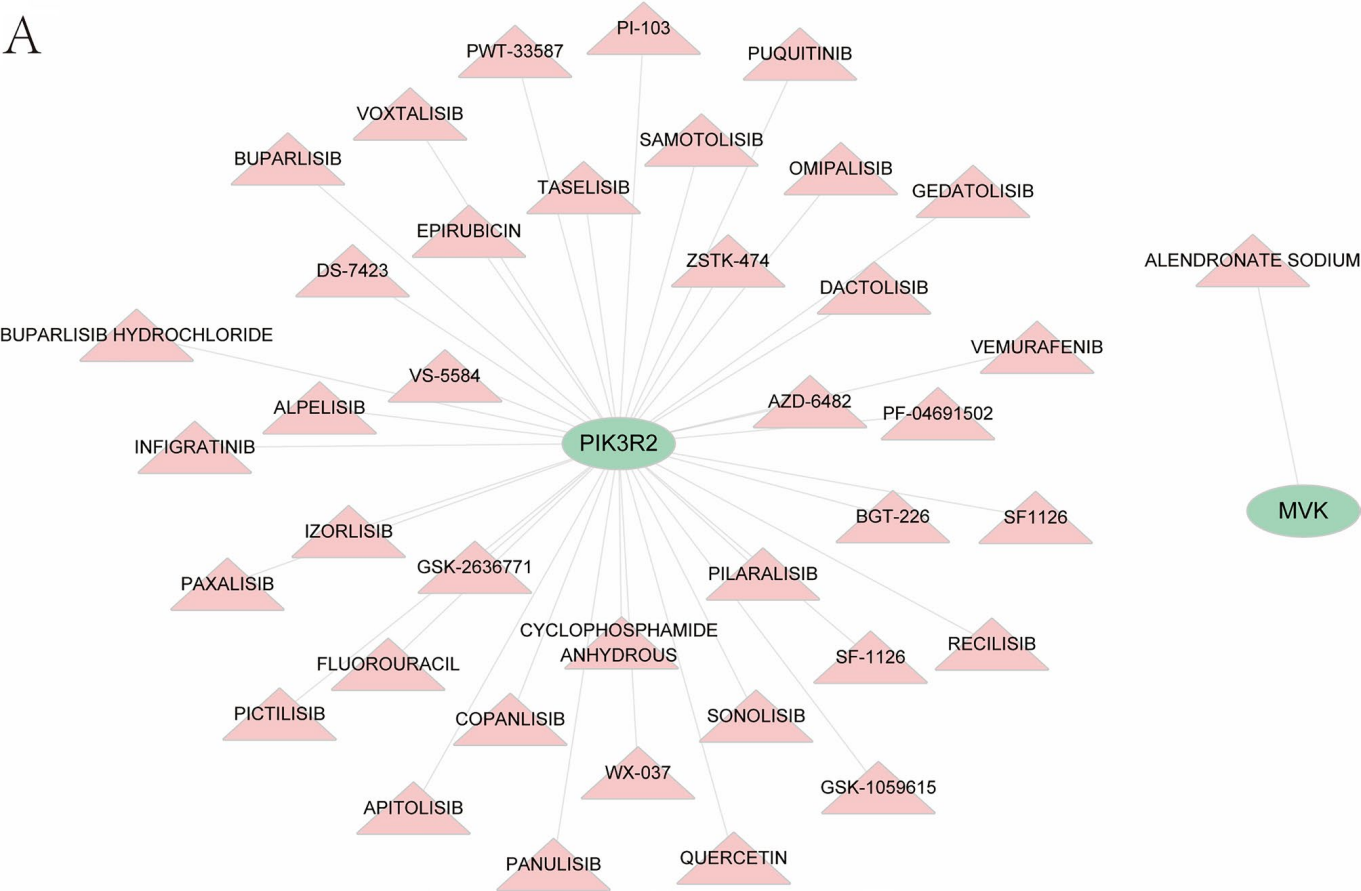
The drugs that interact with three key genes in the DGIdb database.

Using the DGIdb, we identified potential drugs targeting the key genes. The analysis revealed 38 drugs interacting with PIK3R2, offering promising targets for future therapeutic developments (Figure 12).

## 4 Discussion

NAFLD and CAD are major public health problems worldwide. Both diseases are affected by the combined effects of heredity, diet, and environment. Among them, genetic factors have attracted much attention. In recent years, genes such as apolipoprotein C3 (APOC3), leptin receptor (LEPR) and peroxisome proliferator‐activated receptors (PPAR) have been reported to be associated with NAFLD and CAD [4]. Considering the increasing incidence of NAFLD and CAD, finding key genes may provide new ideas for the treatment of NAFLD and CAD.

First, we analyzed NAFLD and CAD to obtain differential genes, and then intersected them with lipid metabolism genes to screen out three key genes, namely GPD1, MVK, and PIK3R2. Glycerol‐3‐phosphate dehydrogenase 1 (GPD1) produces glycerol‐3‐phosphate (G3P), which connects carbohydrate and lipid metabolism and participates in the NADH/NAD cycle [8]. Therefore, abnormal expression of GPD1 may lead to metabolic diseases. Studies have found that GPD1 has a pro‐obesity effect, and the activity of GPD1 is enhanced in morbidly obese patients [9, 10]. There is a correlation between GPD1 expression and obesity, body mass index (BMI) and fat mass. In addition, G3P produced by GPD1 is considered to be the driving force for triglyceride (TG) accumulation [8]. Studies have found that GPD1 gene mutation can lead to hypertriglyceridemia (HTG) [11, 12]. HTG is closely related to NAFLD and CAD and is one of their important risk factors [13]. Among them, the Mevalonate Kinase (MVK) gene plays an important role in cholesterol synthesis [14, 15]. Cholesterol level is one of the risk factors for atherosclerosis, and atherosclerosis can lead to the occurrence and progression of CAD [13]. NAFLD patients also often have abnormal cholesterol levels. For the Phosphoinositide‐3‐Kinase Regulatory Subunit 2 (PIK3R2) gene, studies have found that biotransformed bear bile powder regulates arginine biosynthesis through the FXR/PXR‐PI3K‐AKT‐NOS3 axis to improve diet‐induced NAFLD in mice [16]. In addition, studies have suggested that miR‐126 targets PIK3R2 to inhibit endothelial progenitor cells (EPC) in endothelial mesenchymal transition (EndMT), which may become a therapeutic tool for cardiovascular diseases [17].

In addition, through immune infiltration analysis, it was found that key genes were closely related to the level of immune cell infiltration and played an important role in the immune microenvironment. In NAFLD and CAD, GPD1 is associated with macrophages. Macrophages, as the core cells of innate immunity, play a key role in the occurrence and development of NAFLD and CAD through lipid metabolism, inflammation regulation and other pathways [10, 18, 19]. GPD1 is a key enzyme in lipid metabolism [8], and its interaction with macrophages presents a complex regulatory network in both diseases. Combined with previous studies and our analysis, we believe that GPD1 may act as a “metabolic‐immune” intersection in NAFLD and CAD by regulating lipid metabolism reprogramming of macrophages. Targeting GPD1 (such as small molecule inhibitors) may provide a new strategy for the comorbidity of NAFLD and CAD by reshaping macrophage function and improving metabolic abnormalities and inflammation. We also found that key genes are closely related to lipid metabolism related signatures, which may affect disease changes.

Then, we performed single‐cell sequencing analysis of key genes in NAFLD and CAD, respectively. In NAFLD, GPD1, MVK, and PIK3R2 were found to be more active in cells with high expression of bile acid metabolism in the immune pathway. Previous studies have suggested that bile acid is associated with the occurrence and development of NAFLD and is a potential therapeutic target for NAFLD, especially in the pathways of farnesoid X receptor (FXR) and G protein‐coupled receptor superfamily (TGR5).

Finally, we used key genes to predict targeted therapeutic drugs and found that one drug interacted with MVK and 38 drugs interacted with PIK3R2. This may help to develop new targets for the treatment of NAFLD and CAD.

However, our research also has some limitations. First, our study stayed in database analysis and identified only three key genes, but the molecular mechanism of how they affect NAFLD and CAD is still unclear. Secondly, the drugs we screened need to be verified by further experimental and clinical studies. Therefore, reasonable experiments should be carried out to gradually verify our conjecture.

## Consent

All authors critically reviewed and approved the final manuscript.

## Conflicts of Interest

The authors declare no conflicts of interest.

## Supporting information


**FIGURE S1:** Pathway enrichment analysis of intersection genes.
**FIGURE S2:** For NAFLD patients, the correlation between key genes and different immune factors.
**FIGURE S3:** For CAD patients, the correlation between key genes and different immune factors.
**FIGURE S4:** All the enriched motifs and corresponding transcription factors of key genes.
**FIGURE S5:** (A) In NAFLD, the correlation between key genes and metabolic pathways. (B) In CAD, the correlation between key genes and metabolic pathways.
**FIGURE S6:** (A) NAFLD‐related 12 cell subtypes. (B) Annotations for each cell subtype. (C) Bubble diagram of classical markers of 7 cell subtypes. (D) The histogram of cell proportion corresponding to 7 cell subtypes.
**FIGURE S7:** Co‐expression network of key gene GPD1 and NAFLD disease‐related genes.
**FIGURE S8:** Co‐expression network of key gene GPD1 and NAFLD disease‐related genes.
**FIGURE S9:** Co‐expression network of key gene GPD1 and NAFLD disease‐related genes.
**FIGURE S10:** (A) CAD‐related six cell subtypes. (B) Annotations for each cell subtype. (C) Bubble diagram of classical markers of six cell subtypes. (D) The histogram of cell proportion corresponding to six cell subtypes.
**FIGURE S11:** Co‐expression network of key gene GPD1 and CAD disease‐related genes.
**FIGURE S12:** Co‐expression network of key gene MVK and CAD disease‐related genes.
**FIGURE S13:** Co‐expression network of key gene PIK3R2 and CAD disease‐related genes.

**TABLE S1:** The correlation between three key genes and metabolic pathways in NAFLD.
**TABLE S2:** The correlation between three key genes and metabolic pathways in CAD.

## Data Availability

The authors confirm that the data supporting the findings of this study are available within the article and its supplementary materials.

## References

[1] J. Li , B. Zou , Y. H. Yeo , et al., “Prevalence, Incidence, and Outcome of Non‐Alcoholic Fatty Liver Disease in Asia, 1999‐2019: A Systematic Review and Meta‐Analysis,” Lancet Gastroenterology & Hepatology 4, no. 5 (2019): 389–398. 30902670 10.1016/S2468-1253(19)30039-1

[2] J. P. Ong , A. Pitts , and Z. M. Younossi , “Increased Overall Mortality and Liver‐Related Mortality in Non‐Alcoholic Fatty Liver Disease,” Journal of Hepatology 49, no. 4 (2008): 608–612. 18682312 10.1016/j.jhep.2008.06.018

[3] C. Söderberg , P. Stål , J. Askling , et al., “Decreased Survival of Subjects With Elevated Liver Function Tests During a 28‐Year Follow‐Up,” Hepatology 51, no. 2 (2010): 595–602. 20014114 10.1002/hep.23314

[4] X. L. Li , J. Q. Sui , L. L. Lu , et al., “Gene Polymorphisms Associated With Non‐Alcoholic Fatty Liver Disease and Coronary Artery Disease: A Concise Review,” Lipids in Health and Disease 15 (2016): 53. 26965314 10.1186/s12944-016-0221-8 PMC4785616

[5] S. Wu , F. Wu , Y. Ding , J. Hou , J. Bi , and Z. Zhang , “Association of Non‐Alcoholic Fatty Liver Disease With Major Adverse Cardiovascular Events: A Systematic Review and Meta‐Analysis,” Scientific Reports 6 (2016): 33386. 27633274 10.1038/srep33386 PMC5026028

[6] N. N. Than and P. N. Newsome , “A Concise Review of Non‐Alcoholic Fatty Liver Disease,” Atherosclerosis 239, no. 1 (2015): 192–202. 25617860 10.1016/j.atherosclerosis.2015.01.001

[7] G. C. Farrell , V. W. Wong , and S. Chitturi , “NAFLD in Asia—As Common and Important as in the West,” Nature Reviews Gastroenterology & Hepatology 10, no. 5 (2013): 307–318. 23458891 10.1038/nrgastro.2013.34

[8] S. Oh , X. L. Mai , J. Kim , A. C. V. de Guzman , J. Y. Lee , and S. Park , “Glycerol 3‐Phosphate Dehydrogenases (1 and 2) in Cancer and Other Diseases,” Experimental & Molecular Medicine 56, no. 5 (2024): 1066–1079. 38689091 10.1038/s12276-024-01222-1 PMC11148179

[9] J. Swierczynski , L. Zabrocka , E. Goyke , S. Raczynska , W. Adamonis , and Z. Sledzinski , “Enhanced Glycerol 3‐Phosphate Dehydrogenase Activity in Adipose Tissue of Obese Humans,” Molecular and Cellular Biochemistry 254, no. 1–2 (2003): 55–59. 14674682 10.1023/a:1027332523114

[10] T. Sledzinski , J. Korczynska , E. Goyke , et al., “Association Between Cytosolic Glycerol 3‐Phosphate Dehydrogenase Gene Expression in Human Subcutaneous Adipose Tissue and BMI,” Cellular Physiology and Biochemistry 32, no. 2 (2013): 300–309. 23942261 10.1159/000354438

[11] L. Basel‐Vanagaite , N. Zevit , A. H. Zahav , et al., “Transient Infantile Hypertriglyceridemia, Fatty Liver, and Hepatic Fibrosis Caused by Mutated GPD1, Encoding Glycerol‐3‐Phosphate Dehydrogenase 1,” American Journal of Human Genetics 90, no. 1 (2012): 49–60. 22226083 10.1016/j.ajhg.2011.11.028 PMC3257852

[12] C. Dionisi‐Vici , E. Shteyer , M. Niceta , et al., “Expanding the Molecular Diversity and Phenotypic Spectrum of Glycerol 3‐Phosphate Dehydrogenase 1 Deficiency,” Journal of Inherited Metabolic Disease 39, no. 5 (2016): 689–695. 27368975 10.1007/s10545-016-9956-7

[13] A. Deprince , J. T. Haas , and B. Staels , “Dysregulated Lipid Metabolism Links NAFLD to Cardiovascular Disease,” Molecular Metabolism 42 (2020): 101092. 33010471 10.1016/j.molmet.2020.101092 PMC7600388

[14] W. Chen , J. Xu , Y. Wu , et al., “The Potential Role and Mechanism of circRNA/miRNA Axis in Cholesterol Synthesis,” International Journal of Biological Sciences 19, no. 9 (2023): 2879–2896. 37324939 10.7150/ijbs.84994 PMC10266072

[15] M. P. Fogarty , R. Xiao , L. Prokunina‐Olsson , L. J. Scott , and K. L. Mohlke , “Allelic Expression Imbalance at High‐Density Lipoprotein Cholesterol Locus MMAB‐MVK,” Human Molecular Genetics 19, no. 10 (2010): 1921–1929. 20159775 10.1093/hmg/ddq067 PMC2860891

[16] S. Jiang , X. Wei , Y. Zhang , et al., “Biotransformed Bear Bile Powder Ameliorates Diet‐Induced Nonalcoholic Steatohepatitis in Mice Through Modulating Arginine Biosynthesis via FXR/PXR‐PI3K‐AKT‐NOS3 Axis,” Biomedicine & Pharmacotherapy 168 (2023): 115640. 37806086 10.1016/j.biopha.2023.115640

[17] J. Zhang , Z. Zhang , D. Y. Zhang , J. Zhu , T. Zhang , and C. Wang , “microRNA 126 Inhibits the Transition of Endothelial Progenitor Cells to Mesenchymal Cells via the PIK3R2‐PI3K/Akt Signalling Pathway,” PLoS One 8, no. 12 (2013): e83294. 24349482 10.1371/journal.pone.0083294 PMC3862723

[18] A. Remmerie and C. L. Scott , “Macrophages and Lipid Metabolism,” Cellular Immunology 330 (2018): 27–42. 29429624 10.1016/j.cellimm.2018.01.020 PMC6108423

[19] D. Hu , Z. Wang , Y. Wang , and C. Liang , “Targeting Macrophages in Atherosclerosis,” Current Pharmaceutical Biotechnology 22, no. 15 (2021): 2008–2018. 33480337 10.2174/1389201022666210122142233

