# Supplementary material for: Underlining the Molecular Mechanism of Nonalcoholic Fatty Liver Disease and Coronary Artery Disease in Lipid Metabolism by Combining Multiple Sets of Data Sets
Source: IUBMB Life. 2025 Jul 9;77(7):e70040. doi: 10.1002/iub.70040 (PMC12239705; doi:10.1002/iub.70040)
Supplement: Supplementary file 1 — FIGURE S1: Pathway enrichment analysis of intersection genes. FIGURE S2: For NAFLD patients, the correlation between key genes and different immune factors. FIGURE S3: For CAD patients, the correlation between key genes and different immune factors. FIGURE S4: All the enriched motifs and corresponding transcription factors of key genes. FIGURE S5: (A) In NAFLD, the correlation between key genes and metabolic pathways. (B) In CAD, the correlation between key genes and metabolic pathways. FIGURE S6: (A) NAFLD‐related 12 cell subtypes. (B) Annotations for each cell subtype. (C) Bubble diagram of classical markers of 7 cell subtypes. (D) The histogram of cell proportion corresponding to 7 cell subtypes. FIGURE S7: Co‐expression network of key gene GPD1 and NAFLD disease‐related genes. FIGURE S8: Co‐expression network of key gene GPD1 and NAFLD disease‐related genes. FIGURE S9: Co‐expression network of key gene GPD1 and NAFLD disease‐related genes. FIGURE S10: (A) CAD‐related six cell subtypes. (B) Annotations for each cell subtype. (C) Bubble diagram of classical markers of six cell subtypes. (D) The histogram of cell proportion corresponding to six cell subtypes. FIGURE S11: Co‐expression network of key gene GPD1 and CAD disease‐related genes. FIGURE S12: Co‐expression network of key gene MVK and CAD disease‐related genes. FIGURE S13: Co‐expression network of key gene PIK3R2 and CAD disease‐related genes. [file IUB-77-0-s002.docx]

## **Supplementary Material**


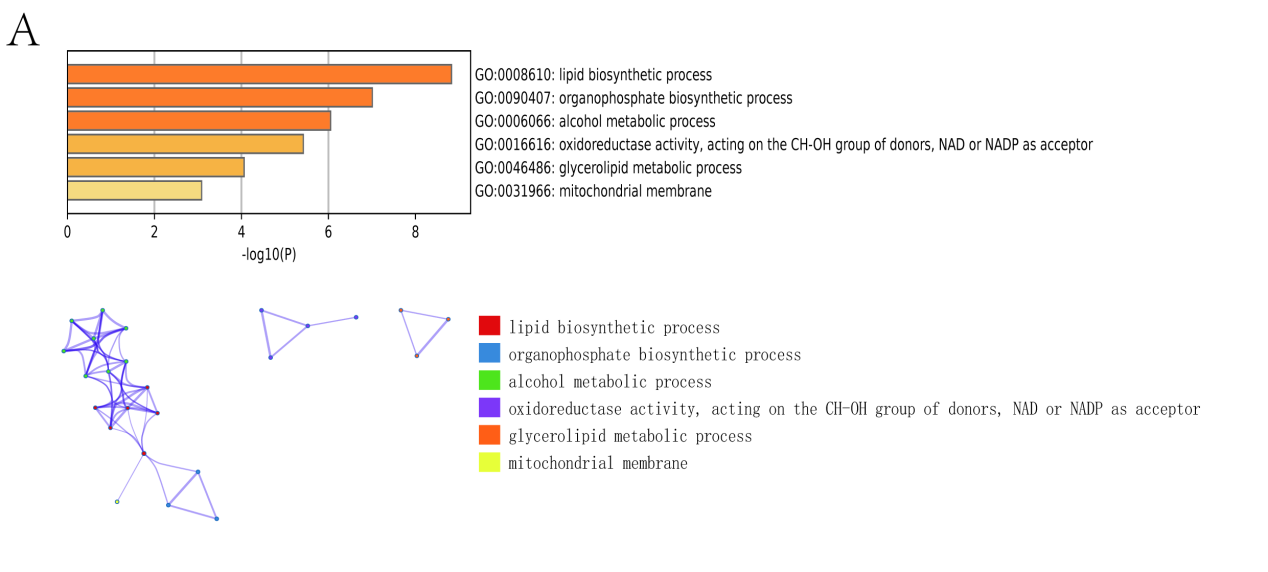


**Figure S1.** Pathway enrichment analysis of intersection genes.


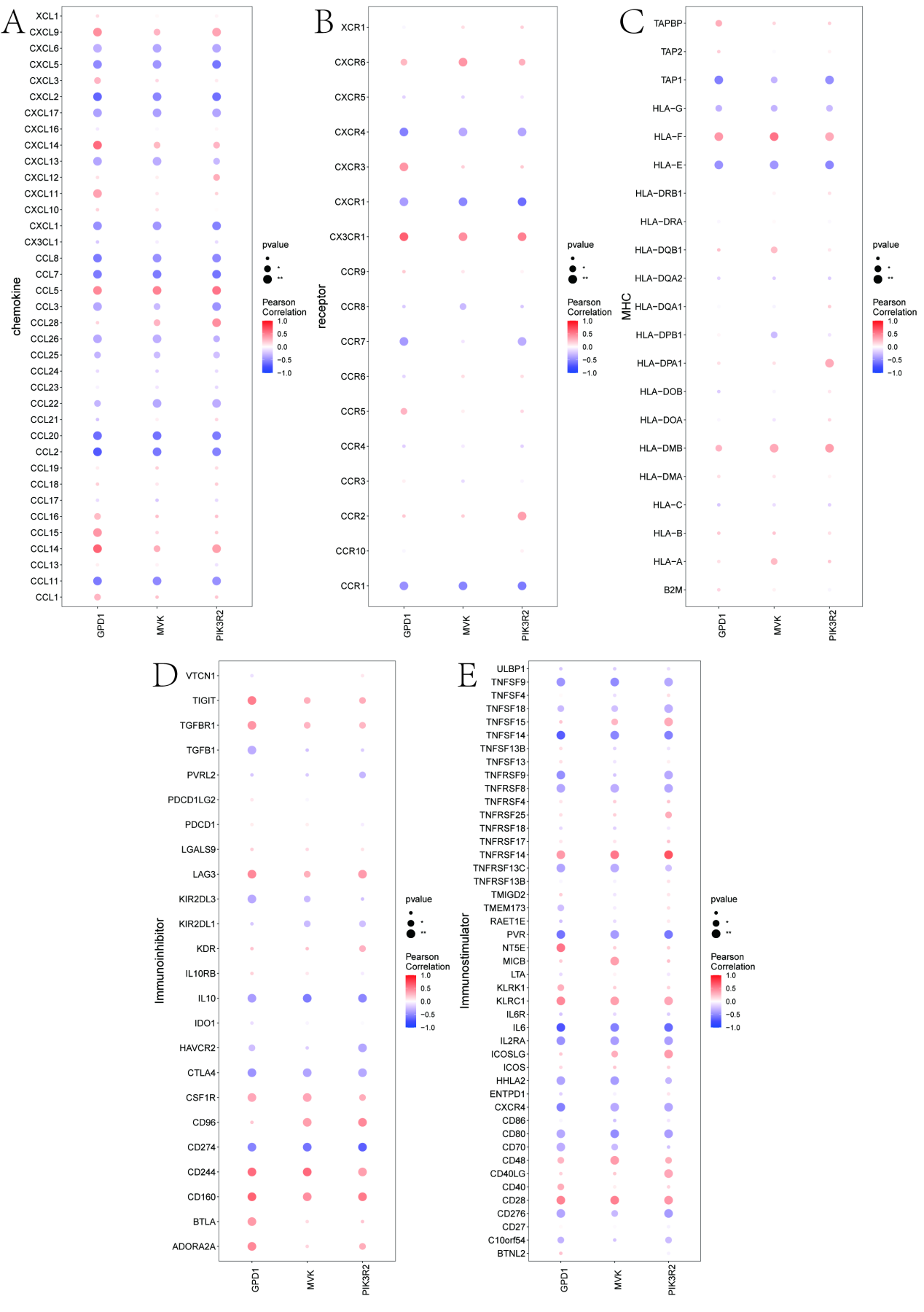


**Figure S2.** For NAFLD patients, the correlation between key genes and different immune factors.


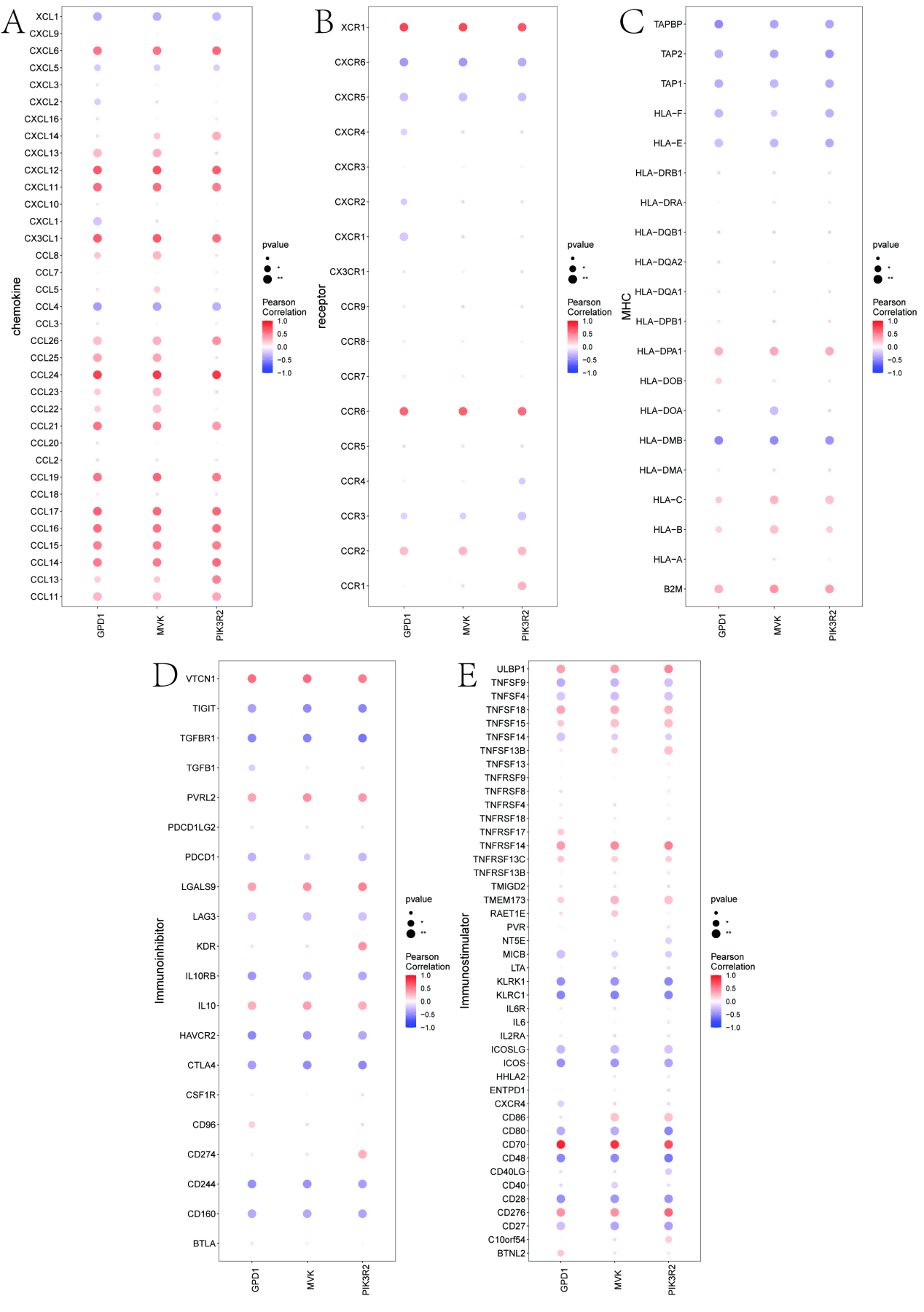


**Figure S3.** For CAD patients, the correlation between key genes and different immune factors.


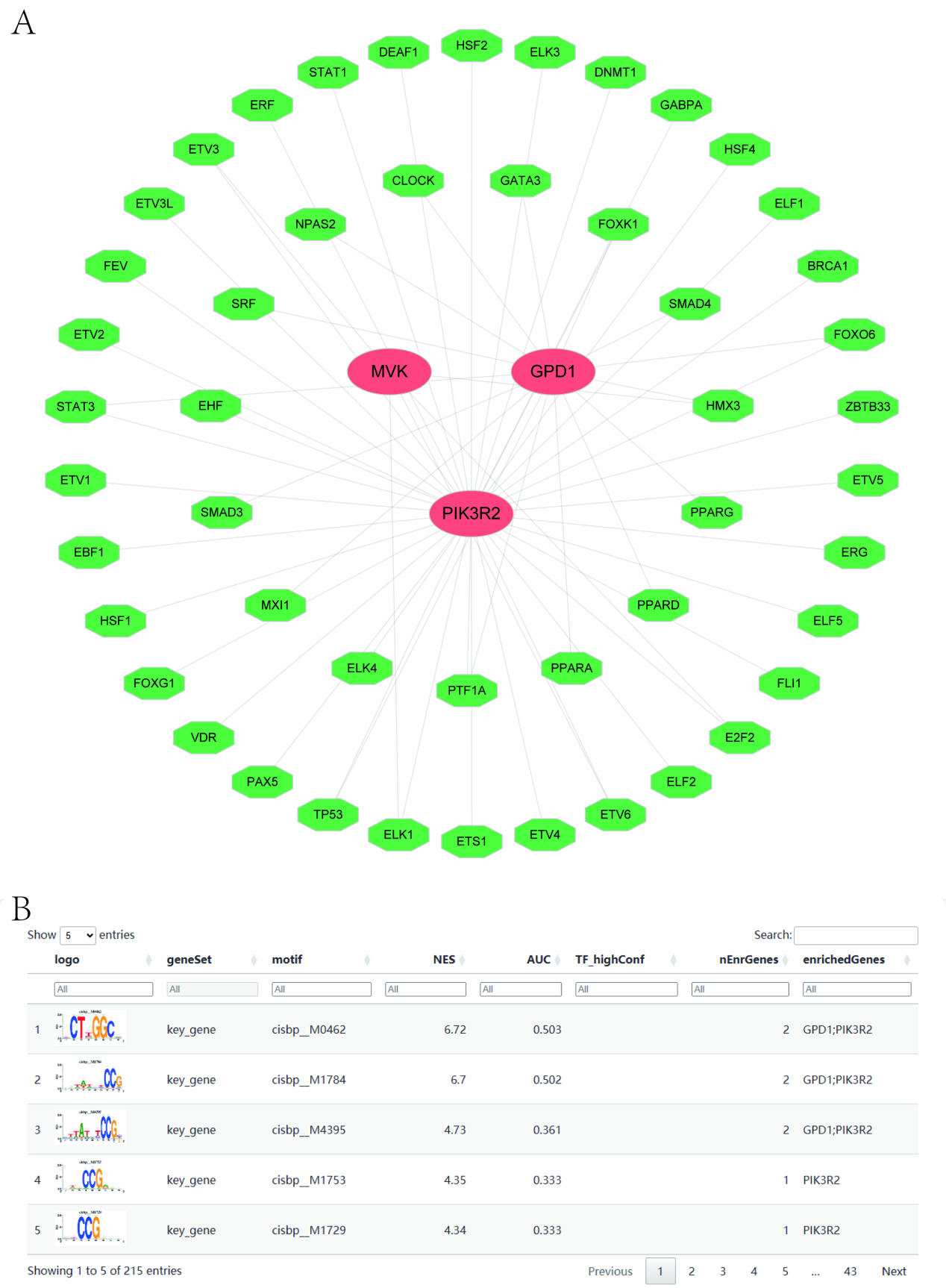


**Figure S4.** All the enriched motifs and corresponding transcription factors of key genes.


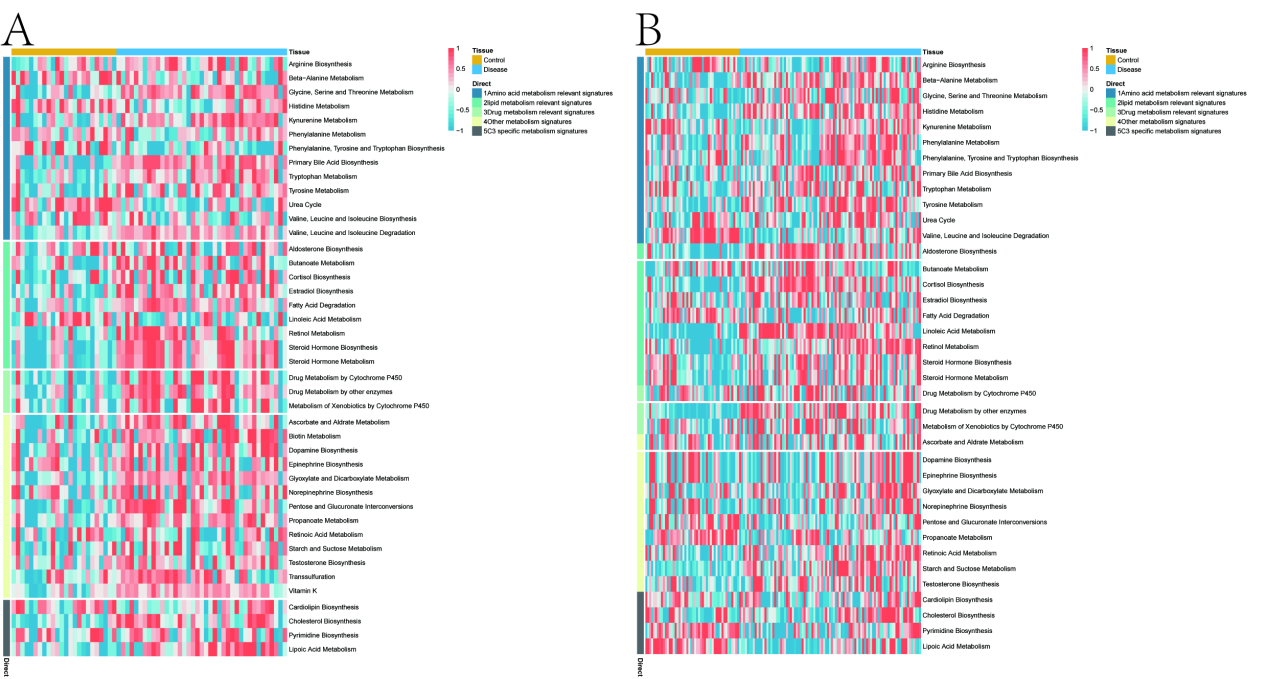


**Figure S5.** In NAFLD, the correlation between key genes and metabolic pathways. B.In CAD, the correlation between key genes and metabolic pathways.


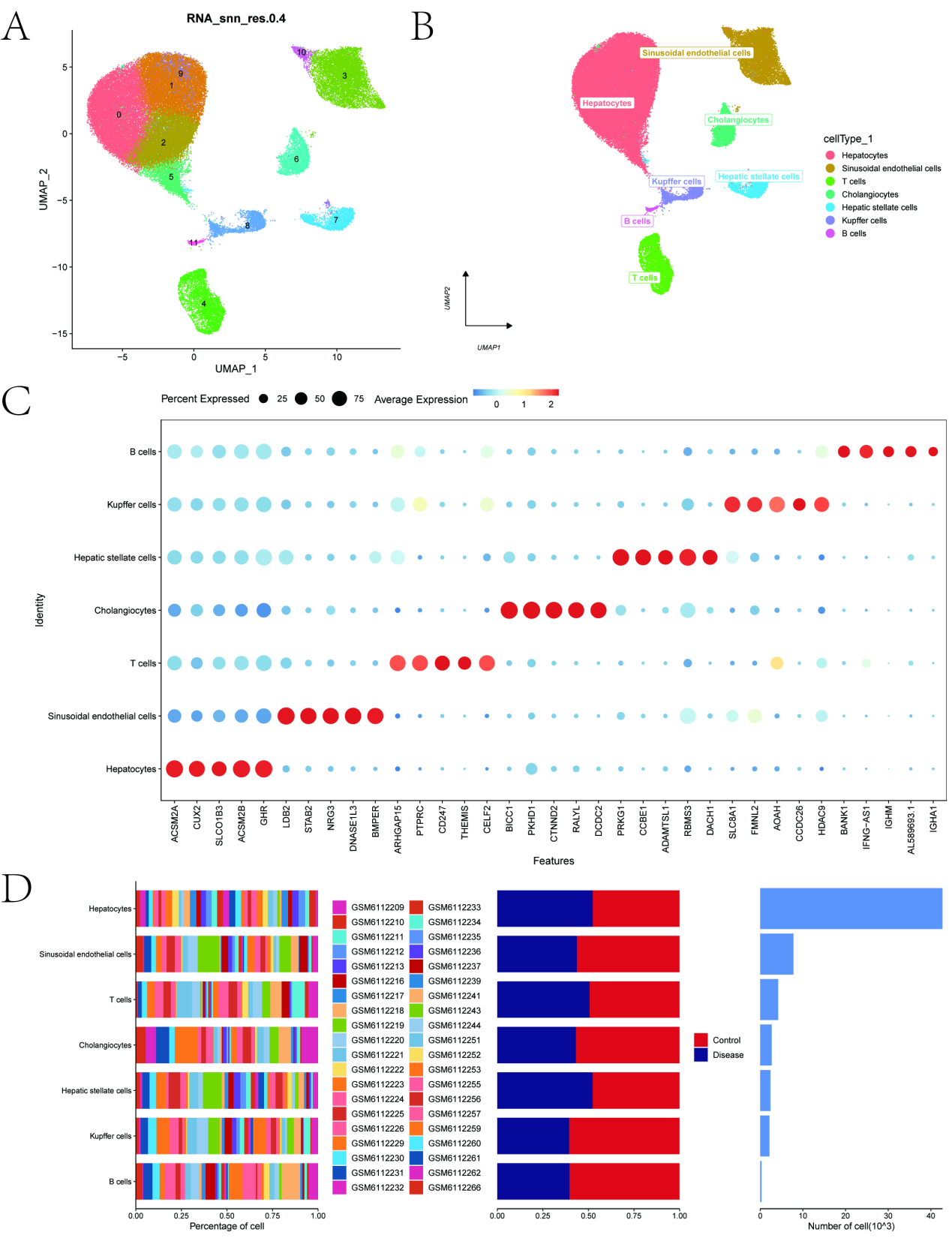


**Figure S6.** A. NAFLD-related 12 cell subtypes. B. Annotations for each cell subtype. C. Bubble diagram of classical markers of 7 cell subtypes. D. The histogram of cell proportion corresponding to 7 cell subtypes.


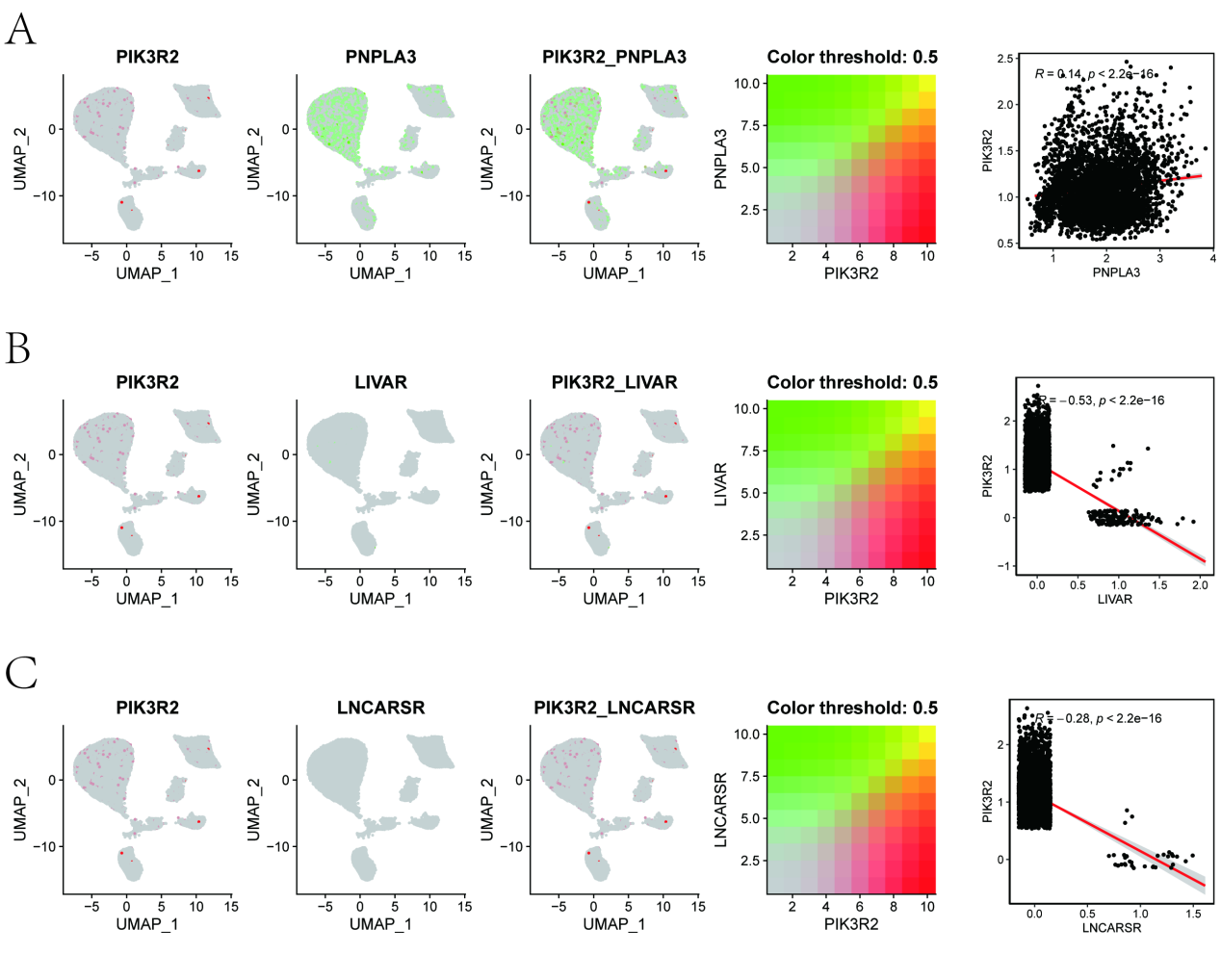


**Figure S7.** Co-expression network of key gene GPD1 and NAFLD disease-related genes.


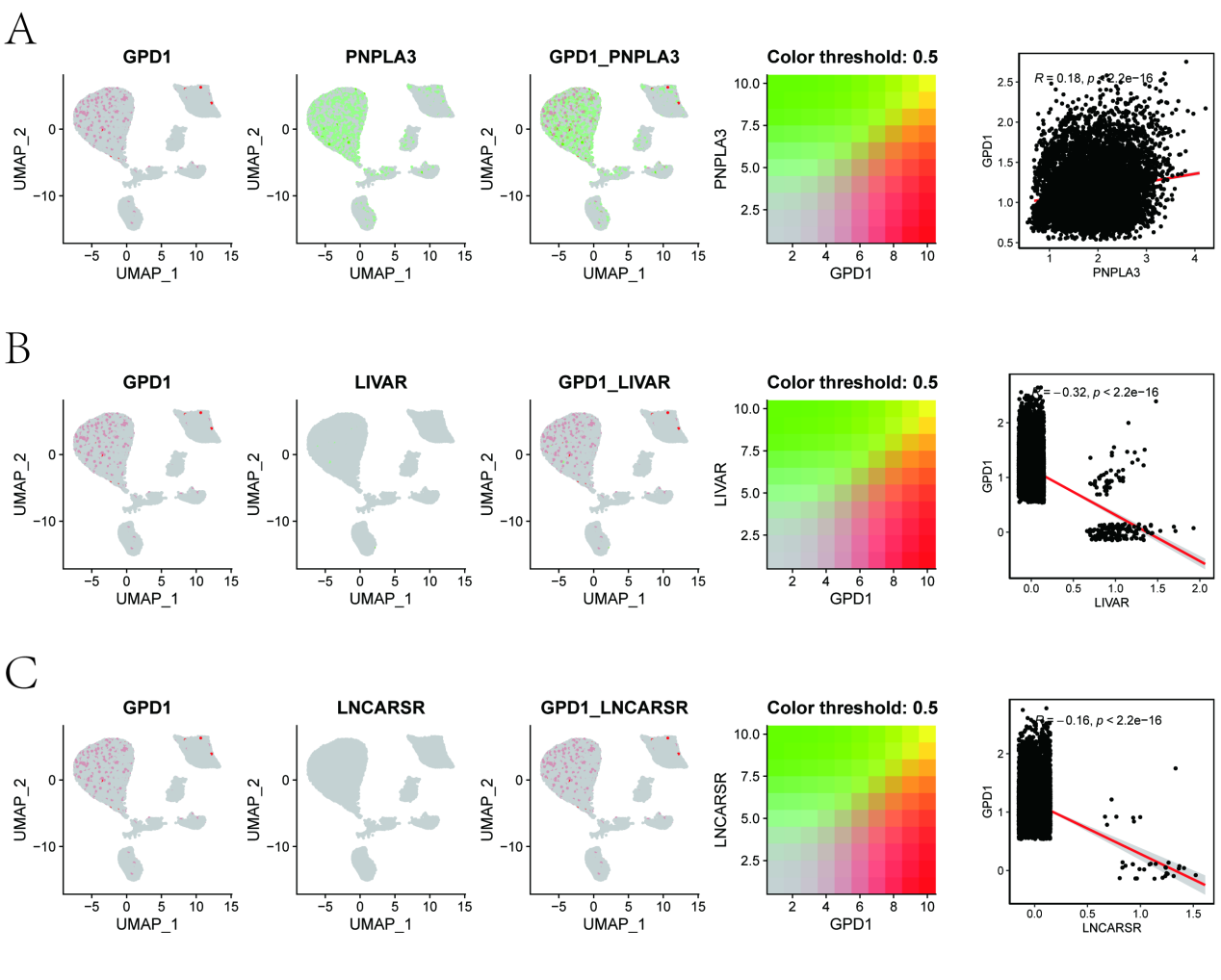


**Figure S8.** Co-expression network of key gene GPD1 and NAFLD disease-related genes.


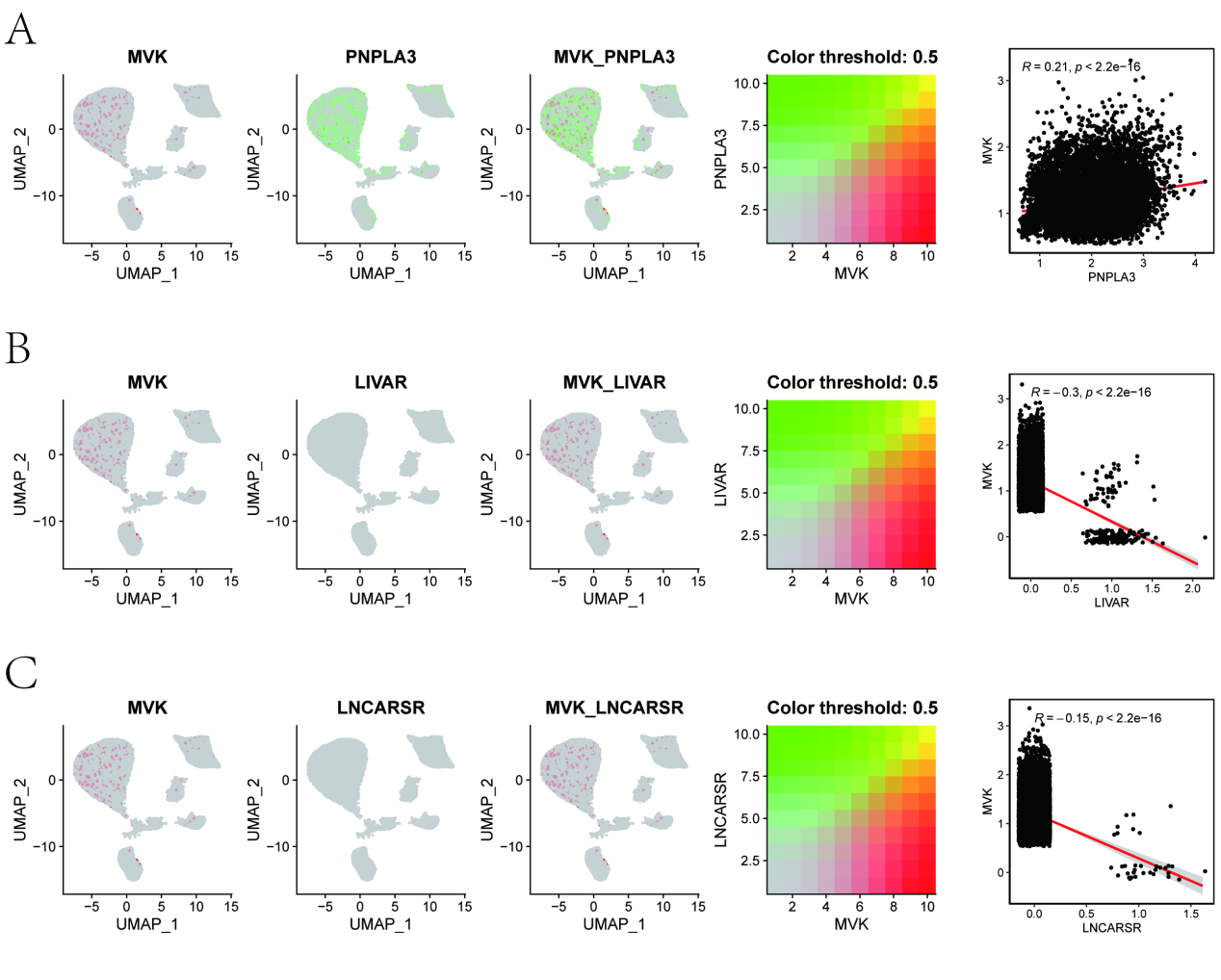


**Figure S9.** Co-expression network of key gene GPD1 and NAFLD disease-related genes.


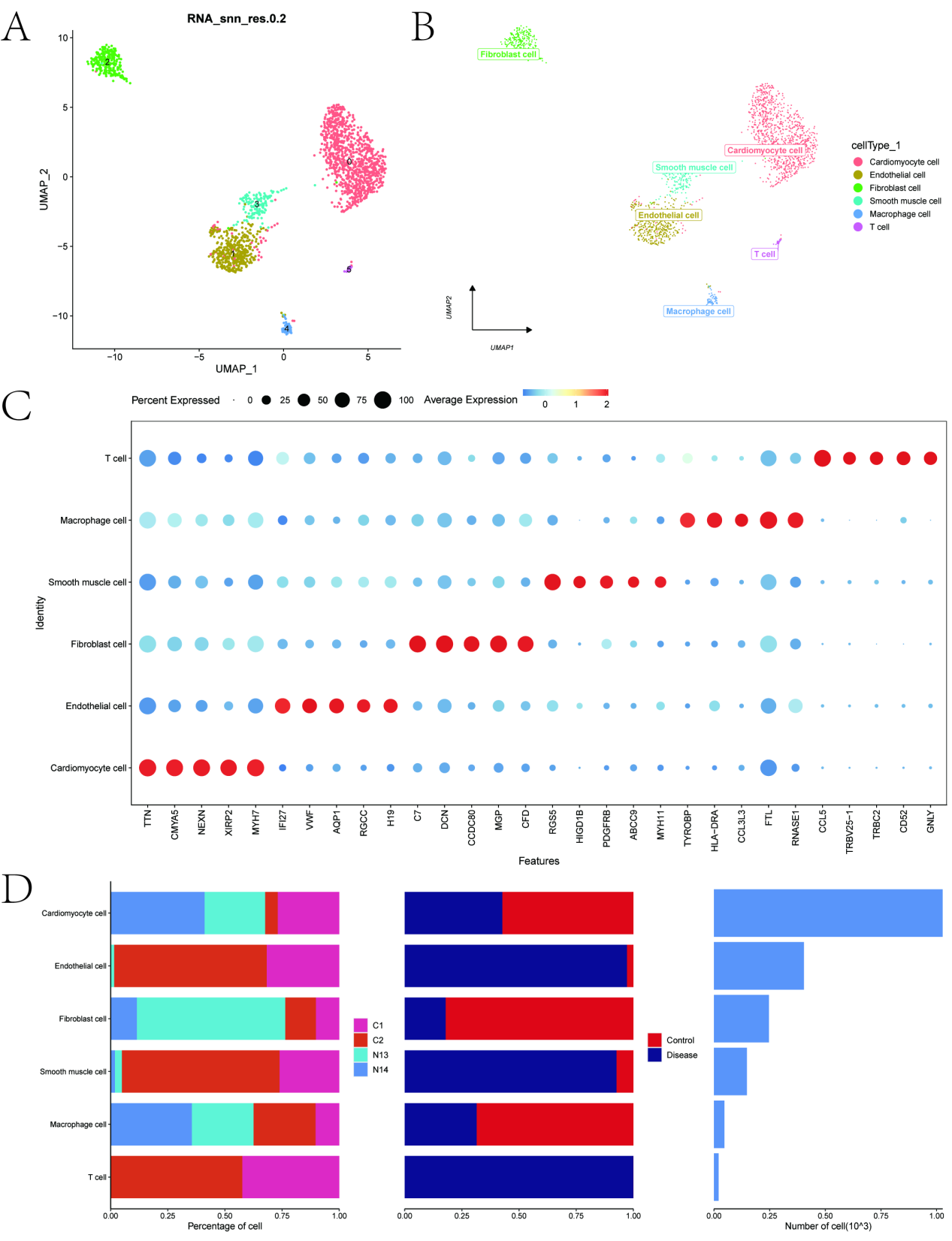


**Figure S10.** A. CAD-related six cell subtypes. B. Annotations for each cell subtype. C. Bubble diagram of classical markers of 6 cell subtypes. D. The histogram of cell proportion corresponding to 6 cell subtypes.


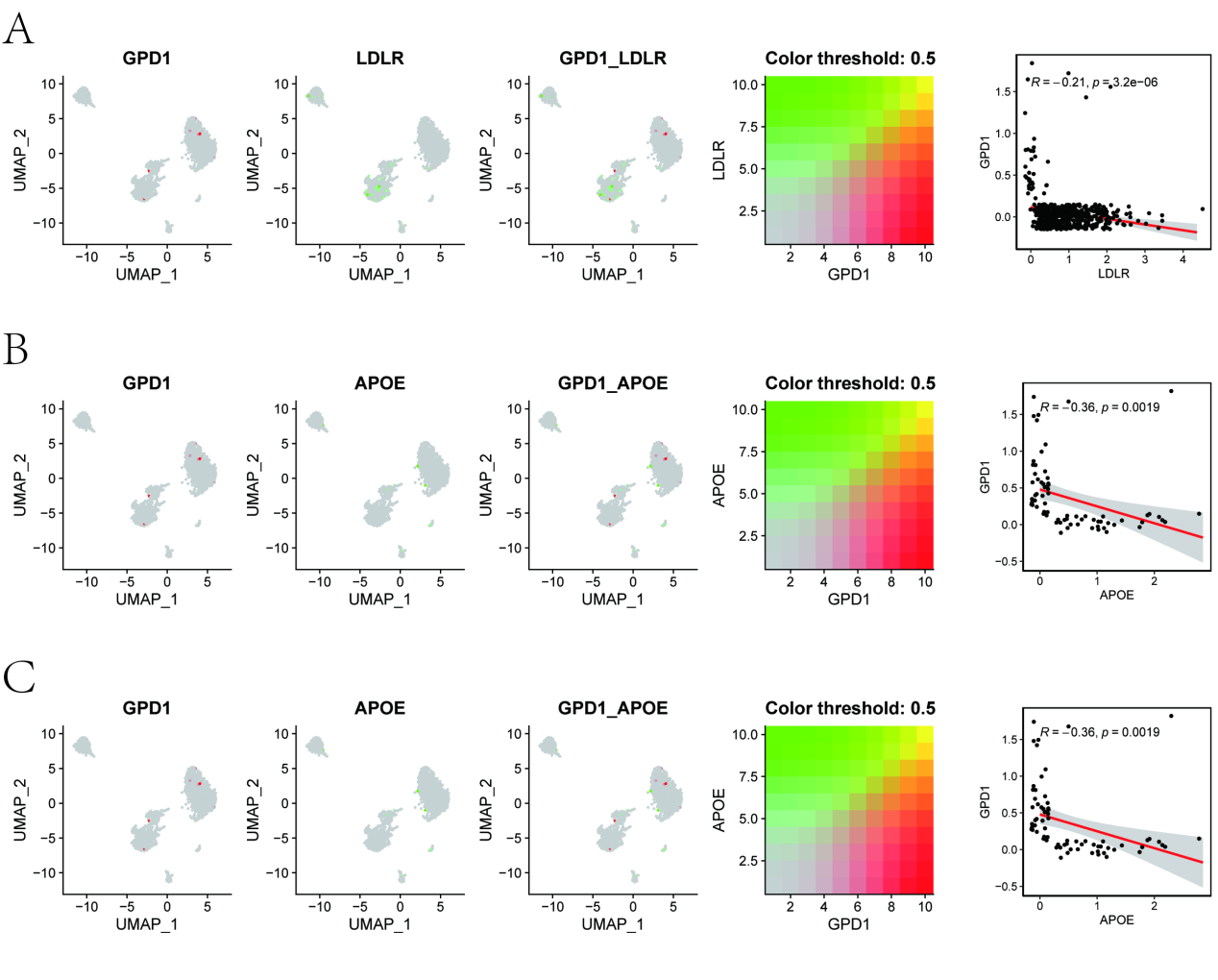


**Figure S11.** Co-expression network of key gene GPD1 and CAD disease-related genes.


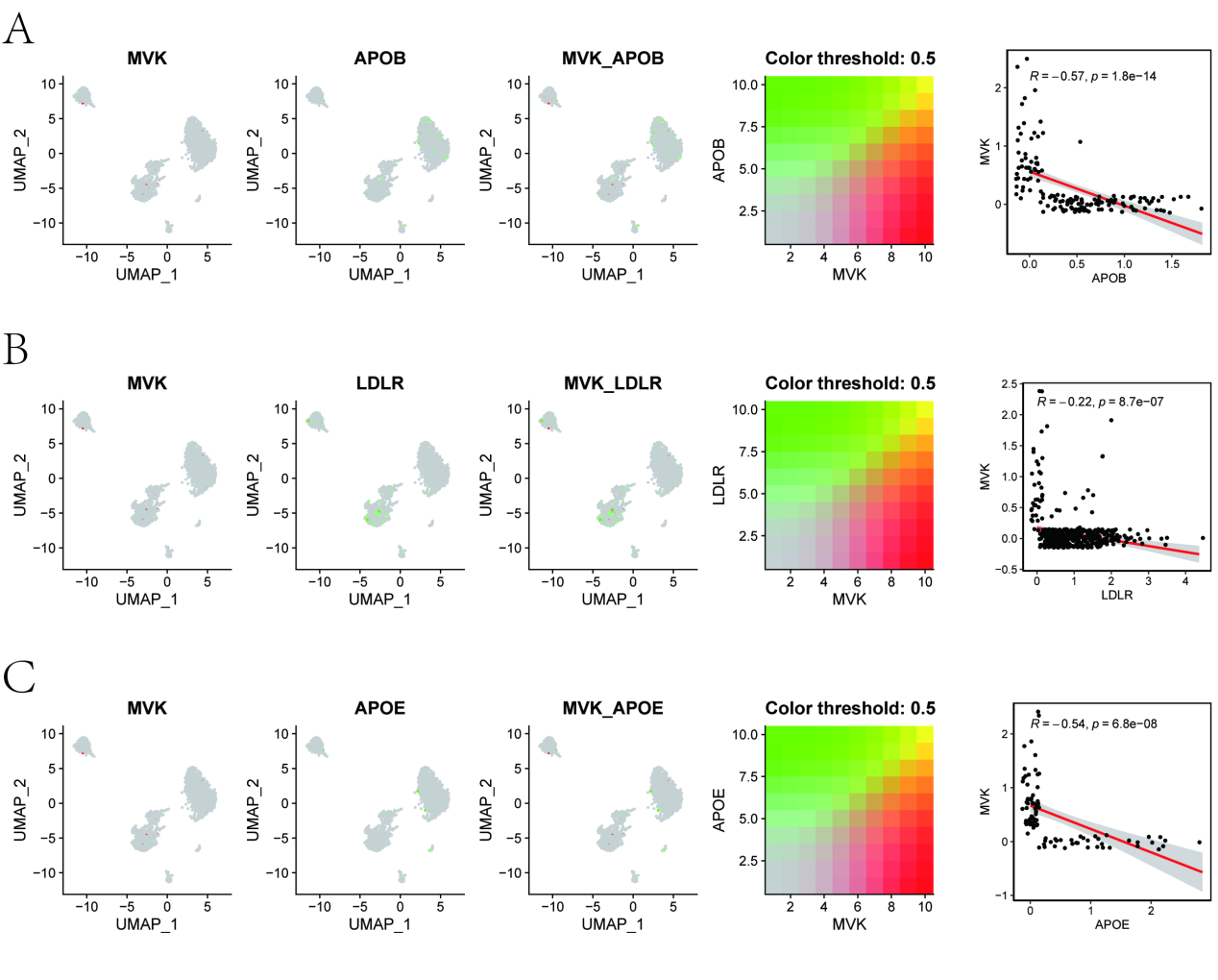


**Figure S12.** Co-expression network of key gene MVK and CAD disease-related genes.


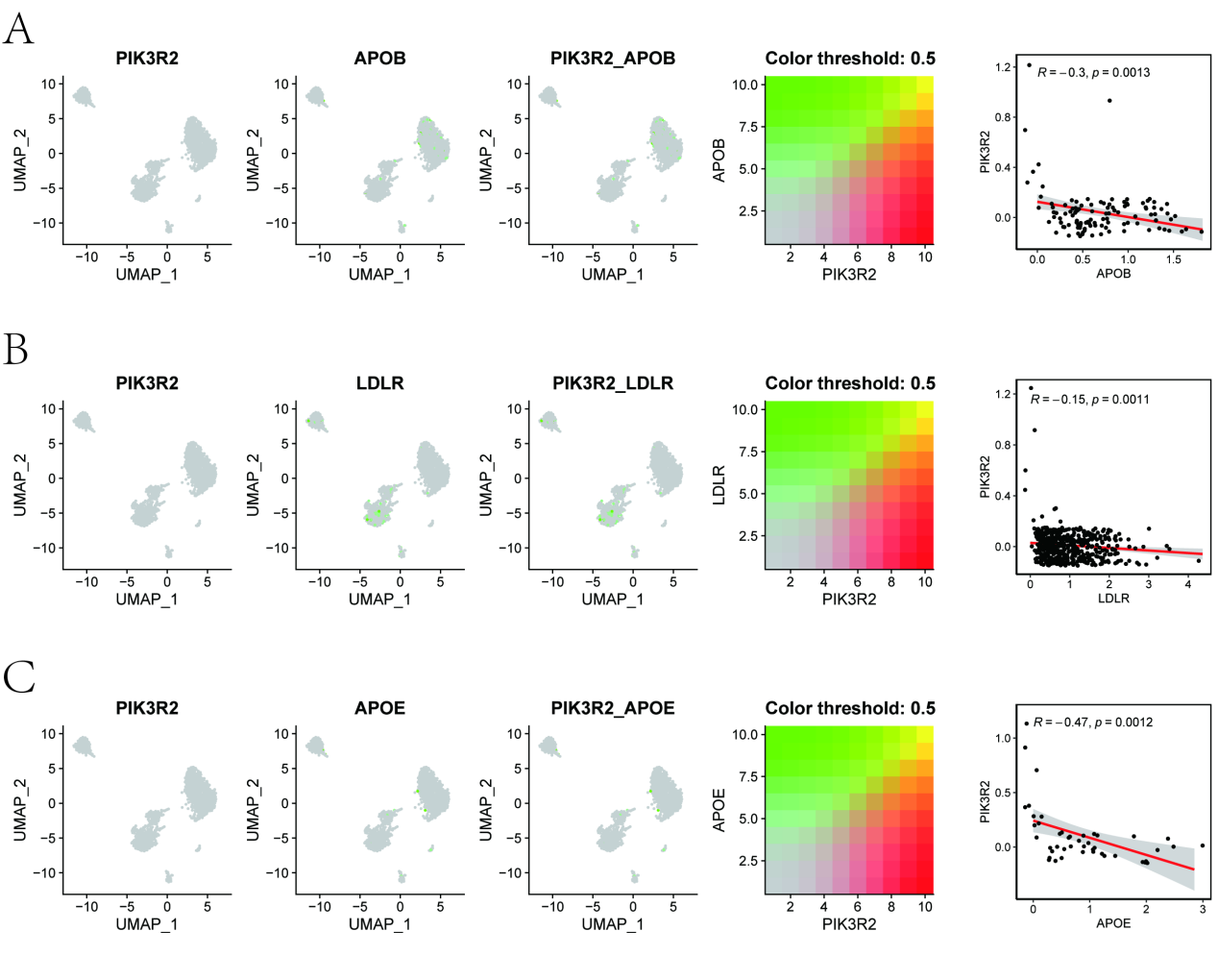


**Figure S13.** Co-expression network of key gene PIK3R2 and CAD disease-related genes.
