# Supplementary material for: Underlining the Molecular Mechanism of Nonalcoholic Fatty Liver Disease and Coronary Artery Disease in Lipid Metabolism by Combining Multiple Sets of Data Sets
Source: IUBMB Life. 2025 Jul 9;77(7):e70040. doi: 10.1002/iub.70040 (PMC12239705; doi:10.1002/iub.70040)
Supplement: Supplementary file 2 — TABLE S1: The correlation between three key genes and metabolic pathways in NAFLD. TABLE S2: The correlation between three key genes and metabolic pathways in CAD. [file IUB-77-0-s001.docx]

**Table S1.** The correlation between three key genes and metabolic pathways in NAFLD.

| Gene1 | Gene2 | Pearson_Cor | Spearman_Cor | CoExp_Cells | Total_Cells |
| --- | --- | --- | --- | --- | --- |
| GPD1 | PNPLA3 | 0.1541 | 0.151 | 9236 | 62229 |
| GPD1 | ADIPOQ | -0.0012 | -6.00E-04 | 2 | 62229 |
| GPD1 | INS | 0.0135 | 0.0173 | 19 | 62229 |
| MVK | PNPLA3 | 0.175 | 0.1762 | 10276 | 62229 |
| MVK | ADIPOQ | 0.008 | 0.0096 | 6 | 62229 |
| MVK | INS | -5.00E-04 | 0.0024 | 11 | 62229 |
| PIK3R2 | PNPLA3 | 0.0392 | 0.046 | 3676 | 62229 |
| PIK3R2 | ADIPOQ | 5e-04 | 3e-04 | 1 | 62229 |
| PIK3R2 | INS | 0.0024 | 0.0037 | 5 | 62229 |

**Table S2.** The correlation between three key genes and metabolic pathways in CAD.

| Gene1 | Gene2 | Pearson_Cor | Spearman_Cor | CoExp_Cells | Total_Cells |
| --- | --- | --- | --- | --- | --- |
| MVK | APOE | -0.0161 | -0.0229 | 0 | 1894 |
| MVK | APOB | -0.02 | -0.0117 | 2 | 1894 |
| MVK | LDLR | 0.0361 | 0.0369 | 16 | 1894 |
| PIK3R2 | APOE | -0.0068 | -0.0096 | 0 | 1894 |
| PIK3R2 | APOB | 0.0378 | 0.018 | 1 | 1894 |
| PIK3R2 | LDLR | -0.0124 | 0.0259 | 4 | 1894 |
| GPD1 | APOE | 0.1178 | 0.0375 | 2 | 1894 |
| GPD1 | APOB | 0.0243 | 0.0508 | 5 | 1894 |
| GPD1 | LDLR | 0.0182 | -0.0185 | 7 | 1894 |
